# Membrane Lipid Composition and Amino Acid Excretion Patterns of *Methanothermococcus okinawensis* Grown in the Presence of Inhibitors Detected in the Enceladian Plume

**DOI:** 10.3390/life9040085

**Published:** 2019-11-14

**Authors:** Ruth-Sophie Taubner, Lydia M. F. Baumann, Thorsten Bauersachs, Elisabeth L. Clifford, Barbara Mähnert, Barbara Reischl, Richard Seifert, Jörn Peckmann, Simon K.-M. R. Rittmann, Daniel Birgel

**Affiliations:** 1 Archaea Physiology & Biotechnology Group, Archaea Biology and Ecogenomics Division, Department of Ecogenomics and Systems Biology, Universität Wien, 1010 Vienna, Austria; ruth-sophie.taubner@univie.ac.at (R.-S.T.); barbara.reischl@univie.ac.at (B.R.); simon.rittmann@univie.ac.at (S.K.-M.R.R.); 2 Institute for Geology, Center for Earth System Research and Sustainability, Universität Hamburg, 20146 Hamburg, Germany; lydia.baumann@uni-hamburg.de (L.M.F.B.); richard.seifert@uni-hamburg.de (R.S.); joern.peckmann@uni-hamburg.de (J.P.); 3 Institute of Geosciences, Department of Organic Geochemistry, Christian-Albrechts-Universität, 24118 Kiel, Germany; thorsten.bauersachs@ifg.uni-kiel.de; 4 Department of Limnology and Bio-Oceanography, Universität Wien, 1010 Vienna, Austria; elisabeth.haberleitner@univie.ac.at (E.L.C.); barbara.maehnert@univie.ac.at (B.M.)

**Keywords:** methanogens, Enceladus, lipids, amino acids, ammonia, formaldehyde, methanol

## Abstract

Lipids and amino acids are regarded as important biomarkers for the search for extraterrestrial life in the Solar System. Such biomarkers may be used to trace methanogenic life on other planets or moons in the Solar System, such as Saturn’s icy moon Enceladus. However, little is known about the environmental conditions shaping the synthesis of lipids and amino acids. Here, we present the lipid production and amino acid excretion patterns of the methanogenic archaeon *Methanothermococcus okinawensis* after exposing it to different multivariate concentrations of the inhibitors ammonium, formaldehyde, and methanol present in the Enceladian plume. *M. okinawensis* shows different patterns of lipid and amino acids excretion, depending on the amount of these inhibitors in the growth medium. While methanol did not show a significant impact on growth, lipid or amino acid production rates, ammonium and formaldehyde strongly affected these parameters. These findings are important for understanding the eco-physiology of methanogens on Earth and have implications for the use of biomarkers as possible signs of extraterrestrial life for future space missions in the Solar System.

## 1. Introduction

Since eons of time, the scientific world has struggled to find a comprehensive and satisfying definition for life. The reason for this could lie in the fact that we know just one sample of life: terrestrial life, also referred to as “life as-we-know-it”. However, only a handful of groups of organic molecules make up the main constituents of biomass of life as-we-know-it. These groups are amino acids, nucleotides, some sugars, fatty acids, and other lipids. This led to the strategy to search for these biomolecules on Earth and other celestial bodies to find indirect evidence for (extraterrestrial) life. In the following, we want to focus on amino acids, i.e., the building blocks of proteins, and isoprenoid-based archaeal membrane lipids as potential biomarkers.

Cell membranes of most terrestrial organisms are glycerol phosphate phospholipids [1]. In Bacteria and Eukarya, they occur in glycerol-3-phosphate configuration, while Archaea use glycerol-1-phosphate to build their polar head groups [2]. It was shown that precursors of lipids like glycerol-1-phosphate can be formed abiotically by prebiotic chemistry by reductive homologation of hydrogen cyanide and some of its derivatives [3]. Other studies demonstrated that glycerophospholipids and other amphiphilic lipids can be abiotically synthesized under early Earth conditions [4]. The integration of lipids into protocell membranes can be regarded as an early evolutionary key step necessary in the emergence of life [5]. All cells have a boundary structure, which separates the interior of the cell from the environment, but at the same time possesses a certain degree of permeability to allow an exchange of nutrients and metabolic by-products. Lipids seem to be perfect for fulfilling this function. As one of many theories about the origin of life, the GARD (Graded Autocatalysis Replication Domain) or “Lipid World” model [5,6,7] postulates that precursors of more complex lipids like short-chain fatty acids originate from meteorites [8] and formed precursor membranes (micelles or vesicles), whereby these assemblies then expanded or split. In other words, lipids or their precursors could have been the first self-replicating entities with “rudimentary life-like properties”, including information storage, in the prebiotic world [5]. Recently, experiments demonstrated that prebiotically plausible amino acids bind to a prebiotically plausible fatty acid, which stabilizes its micelles and vesicles even in the presence of 300 mmol L^−1^ NaCl and 10 mmol L^−1^ Mg^2+^ [9]. This is a possible explanation for two questions. First, the formation of stable lipid vesicles in a salty ocean with divalent cations (fatty acid membranes are considered unstable in solutions >200 mmol L^−1^ NaCl and low mmol L^−1^ concentrations of divalent cations), and second, the coagulation of the precursor molecules of life [9].

Proteinogenic amino acids are the building blocks of proteins. They contain amine (-NH_2_) and carboxyl (-COOH) moieties as functional groups together with a side chain specific to each amino acid. More than 500 naturally occurring species of amino acids are known, but only 20 (plus the amino acids pyrrolysine and selenocysteine that can be incorporated by special translation mechanisms) proteinogenic amino acids are important for building proteins and therefore important for life as-we-know-it [10,11]. In 1953, Miller and Urey showed that proteinogenic amino acids like glycine (Gly), alanine (Ala), and aspartic acid (Asp) might have been produced by the synthesis from simpler organic and inorganic precursors like water (H_2_O), methane (CH_4_), ammonia (NH_3_), and molecular hydrogen (H_2_) that were thought to dominate the primitive atmosphere of the early Earth with the aid of electric discharge, to simulate atmospheric lightning [12]. Other potential sources of abiotic amino acids are submarine hydrothermal systems and extraterrestrial input [13,14]. 

In this study, we demonstrate the effect of potential inhibitors found in the Enceladian plume on the membrane lipid composition and amino acid excretion patterns of a specific methanogenic archaeon (methanogen). Methanogens produce CH_4_ as metabolic end product of their carbon requirement and energy generation and are well known to produce distinct and lasting lipid biomarkers like ether lipids and isoprenoid hydrocarbons, which allow to trace methanogens even when molecular genetic identification is no longer possible [15,16,17]. Some lipids of methanogenic archaea have taxonomic specificity. Methanogens typically exhibit a range of isoprenoid hydrocarbons, such as squalene, diether lipids, and tetraether lipids (e.g., GDGTs: glycerol dialkyl glycerol tetraethers) mostly without cyclopentane or cyclohexane moieties (e.g., [18,19,20,21,22]). In contrast to lipids, virtually nothing is known about amino acid excretion by methanogens. However, it was shown that the methanogenic strains *Methanogenium cariaci* and *Methanococcus thermolithotrophicus* accumulate four different amino acids (L-α-glutamate, β-glutamate, N_ε_-acetyl-β-lysine, and betaine) as a response to osmotic stress [23]. The rare amino acid β-glutamate was also found in the marine strains *Methanocaldococcus jannaschii* JAL-1, *Methanococcus igneus*, and *Methanogenium anulus* AN9 [24].

Methanogens are known to thrive under extreme conditions on Earth. Recent simulation studies demonstrated that species of this group of microorganisms could in principle survive under Mars- or Enceladus-like conditions [25,26,27]. Studies dealing with terrestrial Mars analogues like natural acid streams [28] or extremely dry environments [29] have shown that long-term organic matter preservation is indeed possible in harsh environments, such as Martian soils and rocks. However, icy moons like Europa or Enceladus seem to be far better suited for potentially hosting extraterrestrial life and for the preservation of biomarkers, as complex organic macromolecular material with molecular masses >200 Da was detected [30]. The variety of potential substrates for life (H_2_O, H_2_, CO_2_, etc.) together with the indirect observation of hydrothermal vents at Enceladus’ ocean floor [31] makes this small icy moon, far beyond the traditional habitable zone, one of the hot spots in the search for life in the Solar System.

Many different organic and inorganic molecules were found in Enceladus’ plume [32]. Some of these molecules might inhibit potential methanogenic life on this icy moon. Three methanogenic strains (*Methanothermococcus okinawensis* DSM 14208, *Methanothermobacter marburgensis* DSM 2133, and *Methanococcus villosus* DSM 22612) were tested on their tolerance regarding the potential inhibitors CO and C_2_H_4,_ which were, among other compounds, detected in the plume [27]. *M. okinawensis* was shown not only to just tolerate an environment including the above-mentioned components, but this strain was even able to grow and to release a significant amount of CH_4_ [27], when high amounts of the additional inhibitiors formaldehyde (H_2_CO), ammonium chloride (NH_4_Cl), and methanol (CH_3_OH) were present. In contrast, the other two strains show no or just irregular growth under these harsh conditions. 

H_2_CO, NH_4_Cl, and CH_3_OH can be both inhibitors and precursor molecules of life. The three components are known as constituents of the organic matter of meteorites, comets, and molecular clouds (e.g., [33,34,35,36,37,38,39,40]). H_2_CO, NH_4_Cl, and CH_3_OH were among the first molecules found in the interstellar medium [33,34,36,41,42]. They could have been brought to the young Earth during the Late Heavy Bombardment period about 4–3.85 billion years ago [43,44], when the atmosphere was dense enough to slow down the impactors. Alternatively, they could have been formed directly on Earth or in its early atmosphere [45]. H_2_CO, for instance, could have been produced photochemically by UV irradiation in a CO_2_- and/or CO- and water vapour-containing atmosphere [45,46]. Regardless of whether it was produced on early Earth or it came from extraterrestrial sources, it could have been crucial for prebiotic chemistry. 

These three prominent potential inhibitors NH_4_Cl, H_2_CO, and CH_3_OH were selected to perform an experiment based on a multivariate design space setting (Design of Experiment (DoE)) for testing the influence of these molecules on the growth of *M. okinawensis* [27]. For this DoE, varying amounts of NH_3_ (replaced by NH_4_Cl), H_2_CO, and CH_3_OH in the range of the upper limit of the amount expected to be present on Enceladus were added to the medium.

In the present study, we investigate the effect of inhibitors detected in the Enceladian plume on lipids and excreted amino acids of *M. okinawensis*. These biomolecules could act as biomarkers for methanogenic life on Saturn’s icy moon Enceladus. Here, we do not focus on the existence of single amino acids or lipids, but on a pattern that could be characteristic for life and/or for a specific environmental effect. This may have implications for biomarker search as part of future space missions.

## 2. Materials and Methods

### 2.1. Growth Experiments

Cultivation was performed in 120 mL serum bottles (La-Pha-Pack, Langerwehe, Germany) in chemically defined media with a starting volume of 50 mL (see Taubner et al., 2018 [27], their Supplementary Table S8). The exact procedure of media preparation and inoculation is described elsewhere [27,47]. The inhibitors, NH_4_Cl, H_2_CO, and CH_3_OH, were added to the bottles before autoclaving. The finalization (addition of NaHCO_3_, L-cysteine, and Na_2_S) of the medium and the inoculation was performed in an anaerobic chamber (Coy Laboratory Products, Grass Lake, USA). *Methanothermococcus okinawensis* DSM 14208 was obtained from the *Deutsche Stammsammlung von Mikroorganismen und Zellkulturen GmbH* (DSMZ), Braunschweig, Germany. The injected inoculum was taken from a pre-culture in exponential phase and made up 1% of the total volume. A H_2_/CO_2_ test gas mixture (20 Vol.% CO_2_ in H_2_) of approximately 2 bar relative pressure was applied after each sampling. Sampling (each time approx. 0.75 mL per bottle for optical density (OD) measurement), head space gas pressure measurement, and exchanging the gas phase was done twice a day, approximately every twelve hours. 

Growth was recorded via OD (λ = 578 nm, blanked with Milli-Q water, spectrometer: DU800, Beckman Coulter, USA) and H_2_/CO_2_ to CH_4_ conversion and turnover rate (The turnover rate is equal to the conversion rate per hour of incubation. It was determined via the decrease in headspace pressure in each bottle after each incubation period.) via head space pressure measurements of the serum bottles (digital manometer LEO1-Ei, −1 … 3 bar, Keller, Germany). A zero control was incubated together with the other bottles as background reference for the OD and amino acids measurements. The serum bottles were incubated in the dark in a shaking water bath (65 ± 1 °C). For each experiment, the incubation period between two sampling events was 10.0 ± 0.5 h with a total incubation time of 101 h.

After finishing each experiment, biomass and supernatant were harvested. For this, each culture was centrifuged for 20 min at 4500 rpm (3328 rcf) and 4 °C in 50 mL Greiner tubes (Hettich Universal 320R). The cell pellets and 3 mL of supernatant of each experiment were then separately stored in sterile Eppendorf tubes until further analysis at −20 °C.

#### 2.1.1. Design of Experiment (DoE)

**Figure 1:**
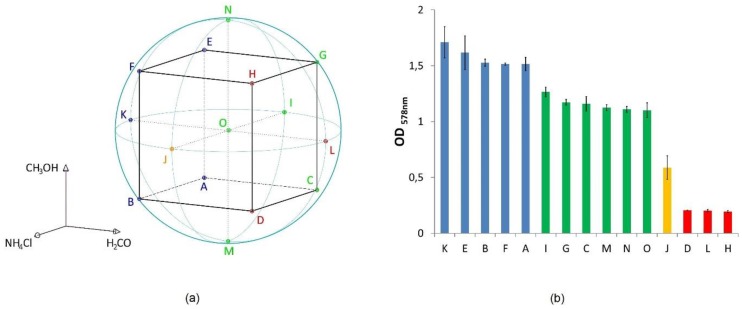
(**a**): Illustration of the central composite design for the Design of Experiment (DoE). For the exact concentration of the three inhibitors at the different points tested, see Taubner et al., 2018 [27], their Supplementary Table S1. The colors are in relation to the grouping observed in the OD_max_ bar chart; (**b**) OD_max_ bar chart of the different datapoints (n = 2 for “F”, n = 3 for all other points, mean values are shown, error = standard deviation).

A central composite design was chosen with a spherical design space with a normalized radius equal to one. Figure 1a shows the theoretical setting of the DoE experiment and Figure 1b shows the maximum optical density (OD_max_) of the respective cultures. All points displayed here are positioned on the sphere—only the central point “O” lies within the sphere. For each of these points, experiments with triplicates were performed and for point “O” even quintuplicates. The concentration of NH_4_Cl, H_2_CO, and CH_3_OH added to the standard medium of *M. okinawensis* for the DoE experiments is given elsewhere (see Taubner et al., 2018 [27], their Supplementary Table S1). Two datapoints of setting “O” and one of setting “F” had to be excluded due to low growth (statistically excluded). Due to fluctuations of the performance of HPLC-APCI-MS (High Performance Liquid Chromatography - Atmospheric Pressure Chemical Ionization - Mass Spectrometry) during lipid analysis, one datapoint of each setting “E”, “J”, and “N” had to be excluded. Further information about the experimental setting is provided elsewhere [27].

#### 2.1.2. Extreme Value Experiments

Here, we tested the growth, amino acid, and lipid production of *M. okinawensis* when exposed to a maximum amount of one certain inhibitor (i.e., 210.91 µL L^−1^ 37% H_2_CO (ROTIPURAN, Roth, Karlsruhe, Germany) (7.656 mmol L^−1^), 210.91 µL L^−1^ CH_3_OH (98%, Sigma-Aldrich, Taufkirchen, Germany) (5.207 mmol L^−1^), or 14.06 g L^−1^ NH_4_Cl (99.5%, Sigma-Aldrich, Taufkirchen, Germany) (0.263 mol L^−1^), respectively) while the other two inhibitors were added at a minimal amount (i.e., 9.09 µL L^−1^ (0.330 mmol L^−1^) H_2_CO, 9.09 µL L^−1^ (0.224 mmol L^−1^) CH_3_OH, and 1.44 g L^−1^ NH_4_Cl (0.027 mol L^−1^), respectively). In the following, the four different settings will be called “Min” (all amounts at minimum), “Me” (high in CH_3_OH), “Am” (high in NH_4_Cl), and “Fo” (high in H_2_CO). The H_2_CO (37 Vol.%) solution also contained minor amounts of CH_3_OH. However, these amounts are as small as the statistical error and are negligible for the current application. All experiments were performed in quadruplicates together with an additional zero control as described in Section 2.1. 

### 2.2. Lipid Extraction and Analysis

The archaeal core lipids were extracted and analyzed as described before [48]. The internal standards 5-α-cholestane (CAS 481-21-0), 1,2-Di-*O*-hexadecyl-*rac*-glycerol (DAGE C_16:16_; CAS 13071-60-8), and 1,2-Di-*O*-octadecyl-*rac*-glycerol (DAGE C_18:18_; CAS 6076-38-6) were added before 0.1 to 15 mg of freeze-dried cells were subjected to acid hydrolysis with HCl (10%) at 110 °C for 2 h. After that, core lipids were extracted with *n*-hexane/DCM (4:1; v:v) to obtain the total lipid extract (TLE). An aliquot of the TLE was subsequently derivatized with acetic anhydride and pyridine for gas chromatography-mass spectrometry (GC-MS) and gas chromatography-flame ionization detector (GC-FID) analyses. GC-MS was used for identification and GC-FID was used for quantification of diether lipids as a control for HPLC-APCI-MS measurements. The GC-MS was a Thermo Scientific Trace GC Ultra coupled to a Thermo Scientific DSQ II mass spectrometer and the GC-FIDs used were a Fisons Instruments GC 8000 series and a Fisons Instruments HRGC MEGA 2 series, both equipped with a flame-ionization detector. The GC columns and the GC temperature program used for the GC-MS and the Fisons Instruments GC 8000 series are described elsewhere [48]. The temperature program used at the Fisons Instruments HRGC MEGA 2 series was as follows: 50 °C for 3 min to 230 °C at 15 °C/min, then held at 230 °C for 2 min and to 325 °C at 6 °C/min and held for 20 min. This modification was due to the limited heating speed of this GC. The retention time of the components was therefore different on the two GC-FIDs, but this did not affect the quantification. The response factor between 5-α-cholestane and DAGE C_18:18_ was 1.6:1 on both GC-FIDs. For identification and quantification of di- and tetraether lipids, 40 µL of a C_46_ GDGT standard (12 mg/L, dissolved in *n*-hexane) was added to an underivatized and unfiltered dry aliquot of the TLE (5%–20% of TLE; injection concentration max. 25 µg/mL) before it was injected into a Varian MS Workstation 6.91 coupled to a Varian 1200 L triple quadrupole MS. The response factors between di- and tetraether lipids were constantly evaluated by injecting a standard mixture after every 4–5 samples [48]. We prepared the standard mixture from synthetic archaeol (1,2-Di-*O*-phytanyl-*sn*-glycerol; CAS 99341-19-2), DAGE C_18:18_, DAGEs C_18:18_-4ene (1,3-Dilinoleoyl-*rac*-glycerol; CAS 15818-46-9), and the aforementioned C_46_ GDGT (CAS 138456-87-8). The response factor between archaeol and C_46_ GDGT was usually around 1.5:1 and the response factor between DAGE C_18:18_ and C_46_ GDGT was between 1.5:1 and 2:1.

### 2.3. Amino Acid Analysis

Prior to analysis, samples were diluted in Milli-Q water at a ratio of 1:4. Analyses were conducted in technical triplicates on an Agilent 1260 Infinity Bioinert HPLC system, consisting of an autosampler, a quaternary pump, a column oven and a fluorescence detector. Derivatization was performed in a robotic autosampler. To 1 mL sample, 75 µL borate buffer (0.4 N in water, pH = 10.2; Agilent Technologies) were added and subsequently mixed. Thereafter, 5 µL OPA reagent (10 mg mL^−1^ of o-phthalaldehyde (OPA) and 3-mercaptopropionic acid in 0.4 mol L^−1^ borate buffer; Agilent Technologies) were added and subsequently mixed as well. After 2 min of reaction time at 27 °C, 100 µL of the reaction mixture were injected into the HPLC system. The separation of the fluorescent derivatives (primary dissolved free amino acids) was achieved on a Zorbax ECLIPSE AAA column (4.6 × 150 mm, 3.5 µm particle size, Agilent Technologies) with a Zorbax ECLIPSE AAA guard cartridge (4.6 × 150 mm, 5 µm particle size, Agilent Technologies), with the column temperature set at 25 °C and a flow rate of 0.8 mL min^−1^. Excitation and emission wavelengths were 340 nm and 450 nm, respectively. Depending on the expected concentrations gain factors of 8 to 12 were used. 

This method is particularly established for samples, where the ammonia concentration immensely exceeds the Dissolved Free Amino Acids (DFAA) concentrations. The quantification of DFAAs in real matrices with high ammonia concentrations is often complicated due to the excess of sodium and interaction with the stationary phase, resulting in peak broadening. Hence, some of the DFAA cannot be quantified, since they are masked by the ammonia peak. Therefore, we modified the gradient, the injection volume, and eluents [49] to reduce peak tailing and suppress matrix effects as described elsewhere [50]. Tetrahydrofuran was used to generally achieve a better separation of individual components, especially between methionine (Met) and valine (Val). Trifluoroacetic acid was added as an ion-pairing agent to improve peak shape, and thus reduce the broadening. Instead of sodium acetate buffer, sodiumdihydrogenphosphate (NaH_2_PO_4_) buffer was used, also resulting in a better ammonia peak shape. The elution gradient is shown in Table S1 and examples of the separation of the dissolved free amino acids are shown in Figures S1 and S2. For peak identification and quantification, a primary amino acid standard mix (AAS18, Sigma Aldrich) was prepared for each run in different concentrations according to the concentration range of the samples (100 nmol L^−1^ to 15 μmol L^−1^). The five missing amino acids (asparagine (Asn), glutamic acid (Glu), gamma-aminobutyric acid (GABA), taurine (Tau), tryptophane (Trp); Sigma Aldrich) were added to the AAS18 standard mix in known concentrations. 

### 2.4. Statistical Analyses

The DoE experiments were analyzed as reported before [27]. Levene tests and ANOVA analyses were performed for the extreme value experiments using the lawstat package of R [51]. Prerequisites for ANOVA were tested using the Levene test at α = 0.05. 

## 3. Results

### 3.1. Lipid and Amino Acid Production under the DoE Setting

*M. okinawensis* grows in the presence of multivariate concentrations of the potential inhibitors NH_4_Cl, H_2_CO, and CH_3_OH [27]. In Figure 1a, an overview of the DoE design space is provided. Figure 1b shows the mean values of OD_max_, an indicator for growth, and Figure S3 shows the corresponding surface plot (dependency on NH_4_Cl, H_2_CO, the combination of NH_4_Cl and H_2_CO, and each parameter squared, see Table S2). The core lipid inventory produced by *M. okinawensis* exposed to the multivariate concentrations of the potential inhibitors is the same as described elsewhere [48], at optimal standard conditions. The lipids comprise archaeol, macrocyclic archaeol, GDGT-0, GTGT-0 (glycerol trialkyl glycerol tetraether), and two isomers of GMGT-0 (glycerol monoalkyl glycerol tetraether) as well as traces of GDD-0 (glycerol dialkyl diether) and GMD-0 (glycerol monoalkyl diether) in some samples [48]. Table 1 shows the respective minimum, mean, and maximum values for different parameters (including lipid and amino acid concentrations, ratio of archaeal di- and tetraethers). The ratio of diethers/tetraethers is defined as (archaeol + macrocyclic archaeol)/ (GTGT + GDGT + GMGTs). Table S3 shows the mean values of each lipid species of all samples of one experiment in percent of total lipids.

All cultures but “D”, “H”, and “L” reached a maximum turnover rate of at least 0.086 h^−1^, whereby the three mentioned ones (all high H_2_CO) stayed below 0.031 h^−1^ for the entire experiment. Overall, the OD_max_ values and the total lipids in ng mg^−1^ dw (dry weight) show a rather similar pattern. The cultures “K”, “E”, “B”, “F” and “A” (all with low H_2_CO amounts) have the highest OD_max_ and “K”, “A”, and “E” show the highest content of lipids. The cultures “D”, “H” and “L” have the lowest OD_max_ and lipid content. Only the cultures “J” seem to be somewhat out-of-line, i.e., it shows a comparatively low OD_max_ compared to other cultures with the same or slightly higher amount of lipids and H_2_CO concentrations (e.g., “C”, “G”, or “I”).

In total, six different lipids are present in all cultures, but just two of them show a statistically significant pattern (GTGT-0 and GMGT-0’, see Figure S4 and Tables S4 and S5). In addition, only the models for the ratios GTGT/GMGT (including both GMGT isomers) and GMGT-0/0’ are significant (see Figure S4 and Tables S6 and S7). The model of GTGT-0 is influenced by CH_3_OH (Table S4), GMGT-0’ by CH_3_OH squared and H_2_CO squared (Table S5), the ratio GTGT/GMGT by H_2_CO and the combination of H_2_CO with NH_4_Cl (Table S6), and the model for the ratio GMGT-0/0’ shows dependency on the combination of H_2_CO and CH_3_OH (Table S7).

By far the most excreted amino acids where found in cultures “I”, whereby cultures “H” showed just one tenth of that amount. The cultures with high NH_4_Cl and medium to high H_2_CO (“H”, “D”, “J”, and “L”) exhibit the lowest amounts of excreted amino acids. Surprisingly, cultures “I” showed the highest amounts of 15 of the 18 measured amino acids, but also showed the lowest amount of glutamine (Gln, 0.40 ± 0.06 µmol L^−1^). The most prominent excreted amino acids found in all cultures were Glu and Gly. In total, 18 different excreted amino acids were detected using the described method, but just 15 of them show a statistically significant pattern (no significant result for histidine (His), Met, and lysine (Lys)). Three patterns are recognizable (see Figures S5–S8): (1) the group Glu, Asn, serine (Ser), Gly, Ala, tyrosine (Tyr), Trp, phenylalanine (Phe) and the combination of all amino acids (“total amino acids”) show a dependence on CH_3_OH squared (see Figure S5 and Tables S8–S16); (2) the amino acids Gln, arginine (Arg), and Val are influenced by the presence of NH_4_Cl (see Figure S6 and Tables S17–S19); (3) the last group containing isoleucine (Ile) and leucine (Leu) are characterized by the influence of NH_4_Cl and CH_3_OH squared (see Figure S7 and Tables S20–S21). The amino acid threonine (Thr) is influenced by the interaction of CH_3_OH and H_2_CO and by CH_3_OH squared (see Figure S8 and Table S22). Asp shows no specific dependency at all, but with a tendency on an influence by CH_3_OH and CH_3_OH squared (see Figure S8 and Table S23).

**Figure 2:**
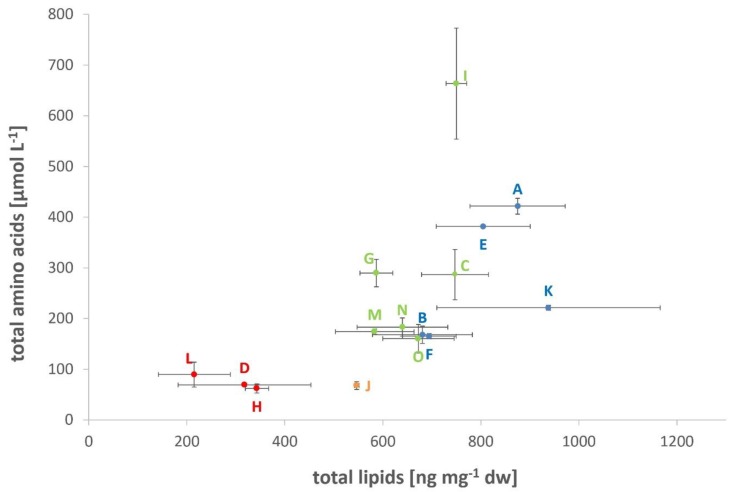
Total lipids [ng mg^−1^ dw] vs. total amino acids [µmol L^−1^]. The colors are in relation to the grouping observed in the OD_max_ bar chart (Figure 1b) (n = 2 for “E”, “F”, “J”, and “N”, all other with n = 3, mean values are shown, error = standard deviation).

In general, the different inhibitors show different impacts on *M. okinawensis* regarding lipid and amino acid production. While the lowest amounts of H_2_CO (cultures “K”) favored growth (OD_max_) and total lipid production, the lowest amounts of NH_4_Cl (cultures “I”) favored total amino acid production (see Figure 2).

### 3.2. Lipid and Amino Acid Production in the Extreme Value Setting

Extreme value experiments served as additional means to evaluate the effect of the different inhibitors. It has to be mentioned that the tested data points do not lie within the design space of the DoE. Levene tests on lipid ratios and all amino acids were performed (see Tables S24 and S25). Table S3 shows the mean values of each lipid species of all samples of one experiment in percent of total lipids. It was not possible to determine the lipid concentrations of the individual samples due to contamination issues of the used standards for this specific experiment. ANOVA (one-way analysis of variance) tests for the ratios of lipids, as well as for each individual excreted amino acid and the total excreted amino acids were performed. For the yield of lipids normalized to the dry biomass, ANOVA tests resulted in significant correlations of H_2_CO to the ratios of diethers/tetraethers, GDGT/GMGT, and GTGT-0/GDGT-0 (see Table S25). For the amino acids, the results of the ANOVA tests can be only presented for Asp. Here, the results indicated that the concentration of NH_4_Cl is the only significant factor affecting the final concentration of Asp at the end of the cultivation (see Table S25). The results of the ANOVA analyses for all other amino acids and the other tested lipid ratios cannot be considered, as the Levene tests were significant for those data (see Table S24).

**Figure 3:**
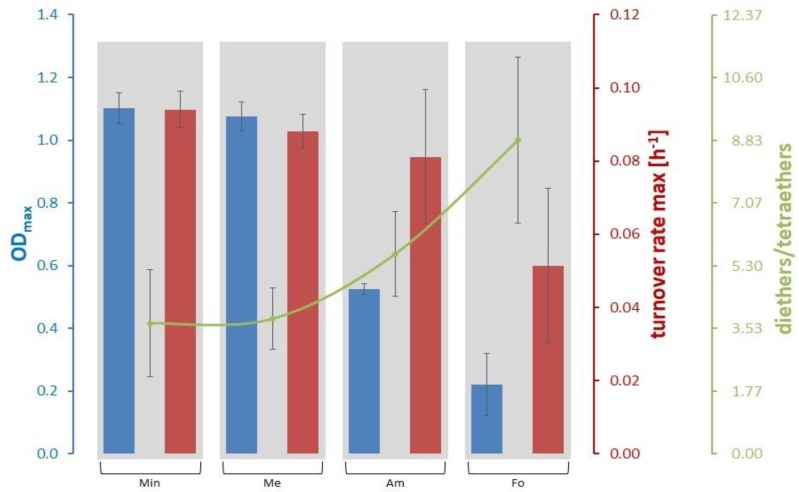
OD_max_, turnover rate max and ratio diethers to tetraethers for the four experimental settings. These are “Min” (all amounts at minimum), “Me” (high CH_3_OH), “Am” (high in NH_4_Cl), and “Fo” (high in H_2_CO). The reason for the chosen order of the four experimental settings in this figure is explained in the second paragraph of Section 3.2.

Trends for growth parameters and lipids are most obvious when putting the conditions into the order “Min”, “Me”, “Am”, and “Fo” which reflects the results of the DoE setting (for abbreviations, see Section 2.1.2). While OD_max_ (for overall growth curves, see Figure S9) and the turnover rate max are decreasing, the ratio of diethers to tetraethers is increasing from left to right (see Figure 3). The same trend can be seen for GTGT/GDGT and a similar one for archaeol/macrocyclic archaeol, GDGT/GMGT, GTGT/GMGT, and GMGT-0/0’. However, in those cases Me shows a slightly lower ratio than Min (see Table 2). The total amount of diethers is almost constant throughout all four experimental settings, but archaeol clearly dominates the ratio archaeol/macrocyclic archaeol for Fo (factor above 6 compared to approx. 1:1 ratio for the other settings, see Table 2). At this high ratio of archaeol/macrocyclic archaeol, also the standard deviation is high. The standard deviations of the ratios GTGT/GDGT and GTGT/GMGT are also higher at condition Fo compared to the other conditions (see Table 2). In contrary, the amount of tetraethers is lowest for Fo and highest for Min, which can be seen in the varying ratio of diethers to tetraethers (Figure 3). The only exceptions for this pattern are the diether archaeol and the tetraether GTGT-0, where Me shows a slightly lower value than Min.

**Figure 4:**
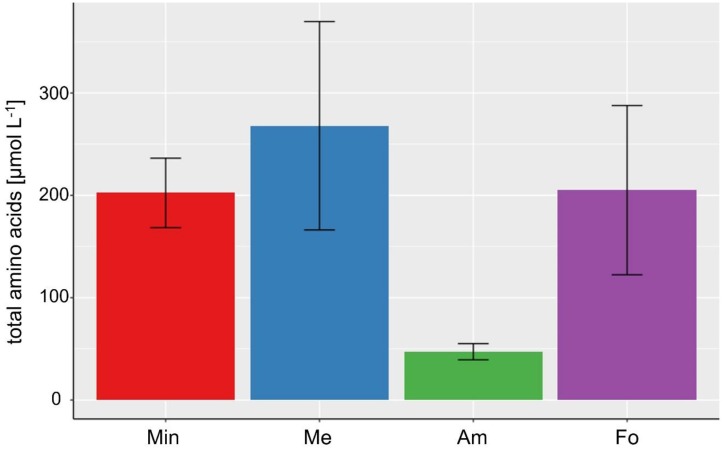
Bar chart of the mean value of the total amino acid concentrations in µmol L^−1^ for the four different experimental settings “Min” (all amounts at minimum), “Me” (high CH_3_OH), “Am” (high in NH_4_Cl), and “Fo” (high in H_2_CO) (n = 4 each, error bars present respective standard deviations). As also seen in the statistical analysis, a high concentration of NH_4_Cl seems to inhibit the amino acid production most strongly.

Contrary to the growth rate and lipid production, and as already seen in the DoE setting, a high amount of NH_4_Cl seems to have the highest negative impact on the amino acid production (see Figure 4 and Table 2). Moreover, the most prominent excreted amino acids are Gly and Glu (see Figure S10). Interestingly, a high amount of CH_3_OH (Me) seems to have a positive impact on the total abundance of amino acids, especially e.g., for Met. In contrast, a high amount of H_2_CO seems to positively affect the production of Gln (although with a high standard variation). While in some of the samples in the DoE study His was detected, no His was detected in any of the samples of the extreme value experiments.

## 4. Discussion

### 4.1. Effect of Varying Physical Conditions and Inhibitors on the Lipid Inventory of M. okinawensis

In the current study, the thermophilic methanogenic archaeon *M. okinawensis* was cultivated at conditions mimicking those potentially occurring on Enceladus, with H_2_ and CO_2_ present, but also with significant amounts of CH_3_OH, NH_4_Cl and H_2_CO. The three latter components are known to inhibit growth and activity of methanogenic archaea, as has been demonstrated previously [27]. Of the strains tested in our previous study [27], only *M. okinawensis* was able to grow under the harsh conditions expected to prevail on Enceladus. In this study, changes in the composition of archaeal lipids and free amino acids were determined under varying concentrations of potential inhibitors for the first time. Lipid production in Archaea at varying temperatures, pressures, pH values, and salinities was studied before (e.g., [52,53]). The first lipid-culture-experiments with the hyperthermophilic, hydrogenotrophic methanogen *Methanocaldococcus jannaschii* were conducted almost 30 years ago, testing either various temperatures or pressures [54,55]. *Methanothermobacter thermautotrophicus* was analysed under changing ambient temperatures [56,57], at different growth stages [58] and its intact polar lipid (IPL) distribution was determined at different growth stages [59] and at varying H_2_ and micronutrient (potassium and phosphate) availability [52]. The closely related *Methanothermobacter marburgensis* was grown with the addition of detergents (polysorbate or Tween^®^-20 or -80, which can lyse mammalian cells [60]). This led to an increased production of GDGT-0 with one additional methyl group in one of the biphytanyl chains. To the best of our knowledge, the present study is the only study investigating core lipids of a methanogen grown in the presence of inhibitors.

The lipid patterns of *M. okinawensis* exposed to DoE concentrations of the three inhibitors and to the extreme concentrations showed distinct, partially unexpected trends. First, the lipid content of the DoE cultures generally followed the population growth curves as determined by OD measurements (Figure 2). This means that the higher the OD_max_, the higher the relative lipid content. H_2_CO had the most significant influence on this ratio. Both OD_max_ and lipid content were highest at the lowest concentrations of H_2_CO and were lowest at its highest concentrations. In contrast, the amount of excreted amino acids was mainly depending on the concentration of NH_4_Cl. The reason for the varying ratio of lipids/biomass and why it correlates with growth are unknown. The lipids/biomass ratio could for instance change when cells change their size. High lipid content would accordingly mean that the cells become smaller. Consequently, cells would become smaller when they grow quicker and are hence dividing faster in our experiments. Possibly, producing less and bigger cells would accordingly be a stress response caused by substrate limitation. As the cell sizes of the cultures were not determined, any further assumptions on that issue cannot be made. Another reason for changes in lipid contents could be the intracellular accumulation of lipids. As shown for a unicellular microalgae strain [61], a bigger population size correlates with a higher lipid content. In addition, the mentioned study found that an increase in cell size, which lowers the surface to volume ratio, leads to a lower lipid content per cell. It needs to be considered that microalgae are photosynthetic eukaryotes, not methanogenic archaea. Therefore, one should be careful drawing conclusions from this comparison. Nevertheless, it could hold true that these basic functions are similar or alike for all cellular, terrestrial organisms.

For both the DoE as well as the extreme value experiments, it seems that the lipids likely make the cell membranes less rigid and more permeable at enhanced concentrations of inhibitors (archaeol and GTGT-0 substitute membrane-spanning, membrane-stabilizing lipids macrocyclic archaeol, GDGT-0, GMGT-0, and GMGT-0’). In archaeol the endings of the alkyl chains are loose, while in macrocyclic archaeol they are connected via a covalent C-C bond. The same is true for GDGT-0 and even more for the GMGTs in comparison with GTGT-0, which has two loose ends of phytane chains. In the GMGTs, a covalent C-C bond links both biphytane chains together, providing higher structural stability. Beside these theoretical considerations, there are studies, which give hints that the content of macrocyclic archaeol and GMGTs indeed increases when temperatures rise [54,62] and the content of GDGT-0 increases when the pH becomes lower [63] (for reviews on that topic, see [53,64]). Assuming the degree of intoxication is increasing with increasing amounts of H_2_CO and NH_4_Cl, these relationships seem to be counterintuitive for the present study. It would imply that *M. okinawensis* makes its membrane more permeable in the presence of—what is commonly seen as—inhibitor for cell growth.

The inhibiting effect of NH_4_Cl and H_2_CO on cell growth was shown before, among others for *M. thermautotrophicus* [65,66]. Therefore, another explanation for the observations of the current study may be that H_2_CO and NH_4_Cl made the cells more permeable by damaging them. One study tested the effect of artificial antimicrobial peptides mimicking those of the immune system of a human gut on methanogens, namely on *Methanobrevibacter smithii*, *Methanosphaera stadtmanae*, and *Methanosarcina mazei* [67]. The peptides affected the pseudomurein walls, the proteinaceous surface layers, and the membranes of the cells. Changes in gene regulation were investigated when *M. thermautotrophicus* was exposed to different environmental stimuli, including 500 mmol L^−1^ NH_4_Cl [66] (in the present study, a maximum amount of 263 mmol L^−1^ was used). This led to an upregulation of several genes related to cell wall modification. Further, it was shown that *M. thermautotrophicus* renders its cells less permeable under nutrient limitation in order to maintain a sufficiently high concentration of the respective nutrient within the cell [52]. This is an example where a methanogen optimized its membrane to challenging environmental conditions. In contrast, the changes in lipid composition of *M. okinawensis* in the current study indicate that the organism does not seem to adapt by producing more resistant lipids (i.e., macrocyclic archaeol, GDGT-0, and GMGTs). It seems that the presence of the inhibitors negatively impacted the production of these lipids in *M. okinawensis*, which are considered to build a more rigid membrane. To assess this effect in more detail, it would be required to identify the biosynthesis routes of ether lipids in *M. okinawensis*. It could be hypothesized that *M. okinawensis* requires more steps to build macrocyclic archaeol, GDGT-0, and the GMGTs than it does to synthesize archaeol and GTGT-0. This leads to the assumptions that archaeol might be the precursor of macrocyclic archaeol or tetraethers and that GTGT-0 might be a precursor in GDGT-0 and the GMGTs synthesis. Since the early 1980s, many studies addressed the question how tetraethers are built and whether diethers are their precursors or not (for review, see e.g., [17,68,69]). The outcomes of these studies are partly contradictory; some studies [70,71,72,73] suggest that macrocyclic archaeol is formed from regular archaeol by adding an additional C-C bond or that GDGT-0 and the GMGTs are formed from either archaeol or GTGT-0 through additional C-C condensations.

### 4.2. Amino Acids and Lipids as Biomarkers for Potential Extraterrestrial Methanogenic Life

Amino acids and (precursors of) lipids might be widely distributed throughout space. In 2003, the detection of Gly in interstellar space was reported [74], which has been highly debated [75]. However, it was later unambiguously found in samples of the comets Wild 2 [76] and 67P/Churyumov-Gerasimenko [77]. In several experiments performed in an environment simulating the interstellar medium (ISM), a large variety of amino acids was synthesized [78]. Moreover, various amino acids were detected in carbonaceous meteorites such as the Murchison meteorite including the proteinogenic amino acids Ser and Thr [79]. Simple fatty acids are the most abundant organic components in carbon-rich meteorites [80]. They were found in various meteorites like the Murchison meteorite (e.g., [80]) or the Tagish Lake meteorite (e.g., [81]). The estimated amount of interplanetary dust particles hitting the early Earth approx. 4.0 Gyr ago was about 10^8^ kg per year [82]. Therefore, extraterrestrial organics likely influenced and triggered the origin of life on Earth.

Recently, it was estimated that hydrothermal H_2_ production of 0.6–34 mol s^−1^ by serpentinization would be enough to sustain abiotic and biotic amino acid synthesis of 0.005–0.25 g s^−1^ and 1–52 g s ^−1^, respectively [83,84]. The resulting concentration of amino acids in a biotic ocean scenario, where methanogens consume virtually all the H_2_, would be max. 90 µmol L^−1^ corresponding to cell concentrations of 80–4250 cells cm^-3^ in the ocean [83]. In comparison, the estimated cell density in the ocean of Europa is 0.1–1 cells cm^−3^ [85]. The amino acid concentration of only hydrothermal-based reactions was calculated to be approx. 104 µmol L^−1^ [83]. These calculations were based on amino acid abundances found in the Murchison meteorite. For the abiotic simulations, Glu, Asp, α- and β-Ala, Gly, and α-amino isobutyric acid (AIB) were used, while for the biotic simulations Glu, Asp, Ala, Ser, and Gly were selected. The latter assumption based on the abundance of amino acids in aquatic ecosystems on Earth [86]. In the present study, a total of 18 amino acids excreted by *M. okinawensis* were detected (Asp, Glu, Asn, Ser, Gln, His, Gly, Thr, Arg, Ala, Tyr, Val, Met, Trp, Phe, Ile, Leu, and Lys). Using the relative abundance of amino acids determined in the current or similar future studies together with those found in the Murray and Murchison meteorites [79,80,87] might help to adapt these in silico simulations. In addition, to distinguish between biotically and abiotically produced amino acids, either the relative abundance compared to Gly could be used or the enantiomeric excess. The latter, however, may not be applicable in the Enceladian ocean, as racemization timescales are short compared to production timescales [83,84]. An alternative approach would be to determine the relative abundance of the detected amino acids to Gly or other prebiotic amino acids, as biosynthetic processes can cause a higher abundance of complex amino acids to optimize protein function and stability [88,89]. Another approach may be the search for specific groups in the side chains of amino acids as biomarkers that are characterized by the highest propensities related to terrestrial life [90]. A recent study showed that amino acids decompose to a great extent on relatively short time scales in oceans with hydrothermal activity [91]. Therefore, detecting amino acids of a concentration higher than 1 nmol L^−1^ on Enceladus, Europa, and other ocean worlds with hydrothermal activity would suggest an active production via geochemical or biotic pathways rather than primordial synthesis [91].

Compared to amino acids, lipids are known to be stable over millions to even billions of years when isolated from oxygen (e.g., [92]). Therefore, they are commonly used to study and reconstruct terrestrial ecosystems over geological time scales [93]. For future space missions, either biological precursors of geolipids or simple lipids (such as fatty acids and short-chain isoprenoids) could act as potential biomarkers to search for extraterrestrial life [93]. More specific information may be obtained by the analysis of the hydrocarbon chain properties of membrane lipids [94]. Lipid biomarkers of possibly methanogenic or methanotrophic archaea have been found in different hydrothermal vent precipitates, such as carbonate chimneys [95], massive sulphides [96,97], vent chimneys made of amorphous silica and barite [98], and also in hydromagnesite-containing serpentinization spring precipitates [99]. Even in old rocks, pristine lipid biomarkers have been successfully assigned to their source. For example, isoprenoid hydrocarbons of methanotrophic archaea have been found in authigenic limestones from the late Pennsylvanian (approx. 300 Ma) in southern Namibia [100] and lipids of methanogenic and halophilic archaea in mid-Neoproterozoic (~820 Ma old) evaporites from central Australia [101]. The lipid biomarker approach may therefore offer an opportunity to search for signs of life in extraterrestrial worlds in the future.

Next generation ground based and orbital telescopes could try to detect biomarkers by using infrared spectroscopy and polarimetry [102]. Analysing the transmission spectra of Europa’s plumes would then be easier than the observation of Enceladus’ plume due to its larger physical scale and shorter distance. The latter observation would be possible when Enceladus crosses the bright disk of Saturn, which will happen again in 2022 [102].

Besides observing Enceladus from Earth, sending a space probe to the icy moons of our Solar System is a main goal for the upcoming decades. There is a variety of promising concepts for future missions to Enceladus, like Enceladus Life Finder (ELF) or Journey to Enceladus and Titan (JET), which were proposed for NASA’s Discovery Mission funding and Enceladus Life Signatures and Habitability (ELSAH) proposed for NASA’s New Frontiers program [103]. On the European side, the Explorer of Enceladus and Titan (E^2^T) was submitted to the ESA M-class call. Together, these missions would perform multi-flybys over the South Polar Terrain of Enceladus. The Life Investigation For Enceladus (LIFE) on the other hand was planned using a sample return concept on a 13.5-year roundtrip trajectory [104]. For Europa, the upcoming missions Europa Clipper (NASA) and JUICE (Jupiter Icy Moons Explorer, ESA) will be leading the way. Scientists all over the world from different fields are working on gaining a better picture of Enceladus and Europa to help building and calibrating the instruments for the future missions [105]. The current study may contribute to this effort.

## 5. Conclusions 

Enceladus (or other icy moons like Europa) may provide a habitable environment for life to develop, the probability that it is really inhabited is quite low [106]. However, organisms that may thrive on icy worlds like Enceladus would have to face—in the view of life as-we-know-it—extreme environmental conditions like high pH, high pressure, varying temperature, and various substances that may potentially inhibit the formation of life. With respect to the latter, inhibitors like NH_4_Cl, H_2_CO, and CH_3_OH were identified on Enceladus. In this study, we demonstrate that the methanogenic strain *M. okinawensis* shows different patterns in its lipid and amino acid synthesis depending on the concentration of these inhibitors. While CH_3_OH showed no critical impact in the tested concentration range (0.224 to 5.207 mmol L^−1^) on neither growth nor lipid or amino acid production rates, the other two inhibitors had a strong influence on both, growth and production rates. H_2_CO (tested concentration range 0.330 to 7.656 mmol L^−1^) showed a significant negative effect on the growth and lipid production of *M. okinawensis*, especially in the presence of excess NH_4_Cl. On the contrary, amino acid synthesis was mainly negatively influenced by an increasing amount of NH_4_Cl (tested concentration range 0.027 to 0.263 mol L^−1^). The relative abundance of the different lipids and amino acids changed throughout the different experimental settings, but the species inventory of lipids and amino acids stayed the same. Studies like the current one could help to calibrate or adapt instruments for future space missions that aim at detecting signs of extraterrestrial life using biomarkers like lipids or amino acids.

## Figures and Tables

**Table 1:** Minimum, mean, and maximum values (incl. standard deviation) of the DoE study for the parameters OD_max_, turnover rate max, diethers [ng·mg^−1^ dw], tetraethers [ng mg^−1^ dw], ratio diethers/tetraethers, total lipids [ng mg^−1^ dw], and total amino acids [µmol L^−1^]. The capital letters in brackets next to the minimum and maximum values indicate the culture (see Figure 1a).

	Minimum Value	Mean Value	Maximum Value
OD_max_	0.20 ± 0.01 (H)	1.07 ± 0.05	1.71 ± 0.14 (K)
Turnover rate max [h^−1^]	0.028 ± 0.003 (L)	0.082 ± 0.002	0.098 ± 0.000 (G)
Diethers [ng mg^−^^1^ dw]	198.41 ± 58.42 (L)	583.96 ± 68.73	883.79 ± 197.54 (K)
Tetraethers [ng mg^−1^ dw]	16.89 ± 15.16 (L)	42.50 ± 15.63	68.36 ± 7.04 (A)
Diethers/tetraethers	11.53 ± 2.14 (H)	15.31 ± 5.10	19.43 ± 7.00 (G)
Total lipids [ng mg^−^^1^ dw]	215.31 ± 73.42 (L)	626.46 ± 78.95	938.11 ± 228.19 (K)
Total amino acids [µmol L^−1^]	62.08 ± 9.82 (H)	228.00 ± 34.27	663.58 ± 129.24 (I)

**Table 2:** Minimum, mean, and maximum values (incl. standard deviation) of the four experimental settings “Min”, “Me”, “Fo”, and “Am” for the parameters OD_max_, turnover rate max, archaeol/macrocyclic archaeol, diethers/tetraethers, GTGT/GDGT, GDGT/GMGT, GTGT/GMGT, GMGT-0/0’, and total amino acids [µmol L^−1^].

	Minimum Value	Mean Value	Maximum Value
OD_max_	0.22 ± 0.10 (Fo)	0.73 ± 0.43	1.10 ± 0.05 (Min)
Turnover rate max [h^−1^]	0.051 ± 0.021 (Fo)	0.079 ± 0.019	0.094 ± 0.005 (Min)
Archaeol/macrocyclic archaeol	0.76 ± 0.20 (Me)	2.23 ± 2.76	6.38 ± 3.96 (Fo)
Diethers/tetraethers	3.68 ± 1.51 (Min)	5.48 ± 2.41	8.83 ± 2.34 (Fo)
GTGT/GDGT	0.03 ± 0.01 (Min)	0.07 ± 0.06	0.16 ± 0.05 (Fo)
GDGT/GMGT	0.83 ± 0.12 (Me)	1.42 ± 0.83	2.67 ± 0.60 (Fo)
GTGT/GMGT	0.02 ± 0.01 (Me)	0.14 ± 0.20	0.44 ±0.17 (Fo)
GMGT-0/0’	1.96 ± 0.09 (Me)	2.18 ± 0.40	2.78 ± 0.58 (Fo)
Total amino acids [µmol L^−1^]	47.22 ± 7.93 (Am)	180.70 ± 94.02	268.08 ± 101.83 (Me)

## Supplementary Materials

The following are available online at https://www.mdpi.com/2075-1729/9/4/85/s1, Figure S1: HPLC-chromatograms of sample and 2.5 µmol L^−1^ standard analysed with method published in Clifford et al. (2017) [53]. Figure S2: HPLC-chromatograms of samples and 5 µmol L^−1^ standard analysed with new method. Figure S3: Results of the statistical analysis of the DoE (OD_max_). Figure S4: Results of the statistical analysis of the DoE (GTGT-0, GMGT-0’, GTGT/GMGT, and GMGT-0/0’). Figure S5: Glu, Asn, Ser, Gly, Ala, Tyr, Trp, Phe, and total amino acids show a dependence on CH_3_OH squared. Figure S6: Gln, Arg, and Val are influenced by the presence of NH_4_Cl. Figure S7: Ile and Leu are characterized by the influence of NH_4_Cl and CH_3_OH squared. Figure S8: Thr is influenced by the interaction of CH_3_OH and H_2_CO and CH_3_OH squared, and Asp shows no specific dependency at all. Figure S9: Average growth curves of the four different experiments. Figure S10: Concentrations of the different amino acids for the four different experimental settings. Table S1: Elution gradient applied for the separation of dissolved free primary amino acids by HPLC. Table S2: Significant model for OD_max_. Table S3: Mean values of each lipid of all samples of one experiment in percent of total lipids. Table S4: Significant model for GTGT-0. Table S5: Significant model for GMGT-0’. Table S6: Significant model for GTGT/GMGT. Table S7: Significant model for GMGT 0/0’. Table S8: Significant model for Glu. Table S9: Significant model for Asn. Table S10: Significant model for Ser. Table S11: Significant model for Gly. Table S12: Significant model for Ala. Table S13: Significant model for Tyr. Table S14: Significant model for Trp. Table S15: Significant model for Phe. Table S16: Significant model for total amino acids. Table S17: Significant model for Gln. Table S18: Significant model for Arg. Table S19: Significant model for Val. Table S20: Significant model for Ile. Table S21: Significant model for Leu. Table S22: Significant model for Thr. Table S23: Significant model for Asp. Table S24: Levene-tests of lipid and amino acid production in the extreme value setting. Table S25: ANOVA analysis of lipid and amino acid species that showed no significance at the performed Levene test.
