# Supplementary material for: Membrane Lipid Composition and Amino Acid Excretion Patterns of Methanothermococcus okinawensis Grown in the Presence of Inhibitors Detected in the Enceladian Plume
Source: Life (Basel). 2019 Nov 14;9(4):85. doi: 10.3390/life9040085 (PMC6958431; doi:10.3390/life9040085)
Supplement: Supplementary file 1 [file life-09-00085-s001.pdf]

Article

Membrane Lipid Composition and Amino Acid Excretion Patterns of *Methanothermococcus Okinawensis* grown in the Presence of Inhibitors Detected in the Enceladian Plume

Ruth-Sophie Taubner [1,#], Lydia M. F. Baumann [2,#], Thorsten Bauersachs [3], Elisabeth L. Clifford [4], Barbara Mähnert [4], Barbara Reischl [1], Richard Seifert [2], Jörn Peckmann [2], Simon-K. M. Rittmann [1] and Daniel Birgel [2,*]

[1] Archaea Physiology & Biotechnology Group, Archaea Biology and Ecogenomics Division, Department of Ecogenomics and Systems Biology, Universität Wien, 1010 Vienna, Austria.
[2] Institute for Geology, Universität Hamburg, 20146 Hamburg, Germany.
[3] Institute of Geosciences, Department of Organic Geochemistry, Christian-Albrechts-Universität, 24118 Kiel, Germany.
[4] Department of Limnology and Bio-Oceanography, Universität Wien, 1010 Vienna, Austria.
[#] R.-S.T. and L.M.F.B. contributed equally to this work.
[*] Correspondence: daniel.birgel@uni-hamburg.de

Received: 8 October 2019; Accepted: 11 November 2019; Published: date

Table S1: Elution gradient applied for the separation of primary dissolved free amino acids by high performance liquid chromatography (HPLC). Eluent A (polar phase): 40 mM NaH_2PO_4 buffer (pH 7.8 adjusted with NaOH pellets in Milli-Q water; Sigma-Aldrich); B (non-polar phase): 1000/100/2—Methanol (HPLC grade, Sigma-Aldrich)/Milli-Q water/Trifluoroacetic acid (HPLC grade, Roth); C: Tetrahydrofuran (HPLC grade; Sigma-Aldrich), total time: 90 min.

Time (min)	A %	B %	C %
0	90	10	0
2	90	7.5	2.5
40	68	30	2.0
42	64	35	1.0
75	35	65	0
77	0	100	0
80	90	10	0
90	90	10	0

Table S2: ANOVA analysis of DoE settings. Significant model for OD_{max}.

OD_{max}
Transform: Square Root
Constant: 0

Source	Sum of Squares	df	Mean Square	F-value	p-value	
**Model**	3.88	5	0.7752	320.51	**< 0.0001**	significant
A-NH_4Cl	0.7727	1	0.7727	319.46	**< 0.0001**	
B- H_2CO (37%)	2.41	1	2.41	996.27	**< 0.0001**	
AB	0.5444	1	0.5444	225.09	**< 0.0001**	
A^2	0.0335	1	0.0335	13.87	**0.0006**	
B^2	0.0966	1	0.0966	39.93	**< 0.0001**	
**Residual**	0.0919	38	0.0024			
Lack of Fit	0.0617	9	0.0069	6.57	< 0.0001	significant
Pure Error	0.0302	29	0.0010			
**Cor Total**	3.97	43				

Fit Statistics

**Std. Dev.**	0.0492	**R^2**	0.9768
**Mean**	0.9833	**Adjusted R^2**	0.9738
**C.V. %**	5.00	**Predicted R^2**	0.9671
		**Adeq Precision**	48.5543

Final Equation in Terms of Actual Factors

$$\mathrm{Sqrt}(OD_{max}) = +0.890366 +0.075335\ NH_4Cl +0.004703\ H_2CO\ (37\%) -0.000685\ NH_4Cl * H_2CO\ (37\%) -0.002394\ NH_4Cl^2 -0.000016\ H_2CO\ (37\%)^2$$

Table S3: Mean values of the OD_{max}, the turnover rate max, and of each lipid in percent of total lipids of all samples of one experiment. For the OD_{max} and the turnover rate max triplicates (n = 3) were used except for "F" (n = 2). Further, for the lipid analysis, one datapoint of each setting "E", "J", and "N" had to be excluded (n=2). Values of extreme values setting are mean values of four samples (n = 4).

DoE

	K	E	B	F	A	I	G	C	M	N	O	J	D	L	H
**OD_{max}**	1.71	1.62	1.53	1.52	1.51	1.27	1.17	1.16	1.13	1.11	1.10	0.59	0.21	0.20	0.20
**Turnover rate max [h^{-1}]**	0.096	0.096	0.096	0.095	0.096	0.098	0.098	0.098	0.096	0.097	0.093	0.086	0.031	0.028	0.029
**Archaeol**	64.5%	68.3%	63.8%	64.4%	63.0%	63.4%	75.4%	72.6%	71.0%	68.7%	72.9%	71.0%	69.1%	74.0%	68.6%
**Macr. Ar.**	29.7%	25.7%	30.2%	28.8%	29.2%	30.3%	19.3%	19.7%	21.4%	25.1%	18.0%	23.7%	23.5%	18.1%	23.3%
**GTGT-0**	0.2%	0.3%	0.3%	0.3%	0.4%	0.3%	0.5%	0.7%	0.4%	0.5%	0.6%	0.4%	0.4%	0.8%	0.4%
**GDGT-0**	3.9%	4.2%	4.1%	4.7%	5.1%	3.7%	3.5%	5.2%	4.7%	3.6%	5.6%	2.7%	4.8%	4.6%	4.5%
**GMGT-0**	1.1%	1.1%	1.1%	1.2%	1.7%	1.6%	0.8%	1.2%	1.8%	1.5%	2.0%	1.6%	1.6%	1.8%	2.2%
**GMGT-0′**	0.5%	0.5%	0.4%	0.5%	0.7%	0.8%	0.4%	0.6%	0.7%	0.6%	0.8%	0.7%	0.6%	0.6%	0.9%

Extreme values setting

	Min	Me	Am	Fo
**OD_{max}**	1.10	1.08	0.52	0.22
**Turnover rate max [h^{-1}]**	0.094	0.088	0.081	0.051
**Archaeol**	33.4%	33.5%	41.6%	73.5%
**Macr. Ar.**	42.3%	44.9%	43.0%	15.5%
**GTGT-0**	0.3%	0.3%	0.4%	1.1%
**GDGT-0**	12.6%	9.7%	7.8%	6.9%
**GMGT-0**	7.6%	7.7%	4.8%	2.1%
**GMGT-0′**	3.8%	4.0%	2.5%	0.8%

Table S4: ANOVA analysis of DoE settings. Significant model for GTGT-0.

GTGT-0
Transform: Inverse Sqrt
Constant: 0

Source	Sum of Squares	df	Mean Square	F-value	p-value	
**Model**	0.1018	1	0.1018	4.52	**0.0392**	significant
C-CH_3OH	0.1018	1	0.1018	4.52	**0.0392**	
**Residual**	0.9912	44	0.0225			
Lack of Fit	0.3624	13	0.0279	1.37	0.2269	not significant
Pure Error	0.6289	31	0.0203			
**Cor Total**	1.09	45				

Fit Statistics

**Std. Dev.**	0.1501	**R^2**	0.0931
**Mean**	0.6682	**Adjusted R^2**	0.0725
**C.V. %**	22.46	**Predicted R^2**	0.0212
		**Adeq Precision**	5.4241

Final Equation in Terms of Actual Factors

1/Sqrt(GTGT 0a) =

+0.576726

+0.000841 CH_3OH

Table S5: ANOVA analysis of DoE settings. Significant model for GMGT-0′.

GMGT-0′
Transform: Inverse Sqrt
Constant: 0

Source	Sum of Squares	df	Mean Square	F-value	p-value	
**Model**	0.2969	6	0.0495	3.22	**0.0115**	significant
A-NH_4Cl	0.0013	1	0.0013	0.0824	0.7757	
B-H_2CO (37%)	0.0220	1	0.0220	1.43	0.2389	
C-CH_3OH	0.0524	1	0.0524	3.41	0.0725	
A^2	0.0332	1	0.0332	2.16	0.1498	
B^2	0.1168	1	0.1168	7.59	**0.0089**	
C^2	0.1397	1	0.1397	9.09	**0.0045**	
**Residual**	0.5997	39	0.0154			
Lack of Fit	0.2195	8	0.0274	2.24	0.0516	not significant
Pure Error	0.3801	31	0.0123			
**Cor Total**	0.8966	45				

Fit Statistics

**Std. Dev.**	0.1240	**R^2**	0.3312
**Mean**	0.5235	**Adjusted R^2**	0.2283
**C.V. %**	23.69	**Predicted R^2**	0.0443
		**Adeq Precision**	5.6268

Final Equation in Terms of Actual Factors

1/Sqrt(GMGT 0a') =	
-0.131956	
+0.048749	NH_4Cl
+0.004564	H_2CO (37%)
+0.005168	CH_3OH
-0.002589	NH_4Cl^2
-0.000019	H_2CO $(37\%)^2$
-0.000021	CH_3OH^2

Table S6: ANOVA analysis of DoE settings. Significant model for GTGT/GMGT (including both GMGT isomers).

GTGT/GMGT
Transform: Base 10 Log
Constant: 0

Source	Sum of Squares	df	Mean Square	F-value	p-value	
**Model**	0.3236	3	0.1079	3.22	**0.0321**	significant
A-NH_4Cl	0.0020	1	0.0020	0.0612	0.8058	
B-H_2CO (37%)	0.1654	1	0.1654	4.94	**0.0317**	
AB	0.2809	1	0.2809	8.39	**0.0060**	
**Residual**	1.41	42	0.0335			
Lack of Fit	0.2739	11	0.0249	0.6817	0.7449	not significant
Pure Error	1.13	31	0.0365			
**Cor Total**	1.73	45				

Fit Statistics

**Std. Dev.**	0.1830	**R^2**	0.1871
**Mean**	-0.7435	**Adjusted R^2**	0.1290
**C.V. %**	24.61	**Predicted R^2**	0.0224
		**Adeq Precision**	5.3544

Final Equation in Terms of Actual Factors

$$\log_{10}(GTGT/GMGT) = -0.403862 - 0.052201\ NH_4Cl - 0.003250\ H_2CO\ (37\%) + 0.000492\ NH_4Cl * H_2CO\ (37\%)$$

Table S7: ANOVA analysis of DoE settings. Significant model for GMGT-0/0'.

GMGT-0/0'
Transform: Power
Constant: 0

Source	Sum of Squares	df	Mean Square	F-value	p-value	
**Model**	0.7319	4	0.1830	3.69	**0.0117**	significant
A-NH_4Cl	0.1631	1	0.1631	3.29	0.0770	
B-H_2CO (37%)	0.0035	1	0.0035	0.0697	0.7931	
C-CH_3OH	0.1317	1	0.1317	2.66	0.1108	
BC	0.4229	1	0.4229	8.53	**0.0057**	
**Residual**	2.03	41	0.0496			
Lack of Fit	0.3604	10	0.0360	0.6681	0.7445	not significant
Pure Error	1.67	31	0.0539			
**Cor Total**	2.76	45				

Fit Statistics

**Std. Dev.**	0.2226	**R^2**	0.2647
**Mean**	2.34	**Adjusted R^2**	0.1930
**C.V. %**	9.50	**Predicted R^2**	0.0608
		**Adeq Precision**	7.0124

Final Equation in Terms of Actual Factors

(GMGT 0a/0a')^1 =

+1.84499

+0.017060 NH_4Cl

+0.004308 H_2CO (37%)

+0.003194 CH_3OH

Table S8: ANOVA analysis of DoE settings. Significant model for glutamic acid (Glu).
Glu
Transform: Inverse Sqrt
Constant: 0

Source	Sum of Squares	df	Mean Square	F-value	p-value	
**Model**	0.0284	4	0.0071	3.64	**0.0130**	significant
B- H_2CO (37%)	0.0022	1	0.0022	1.14	0.2914	
C-CH_3OH	0.0035	1	0.0035	1.79	0.1891	
BC	0.0075	1	0.0075	3.83	0.0577	
C^2	0.0152	1	0.0152	7.78	**0.0081**	
**Residual**	0.0761	39	0.0020			
Lack of Fit	0.0174	10	0.0017	0.8597	0.5788	not significant
Pure Error	0.0587	29	0.0020			
**Cor Total**	0.1044	43				

Fit Statistics

**Std. Dev.**	0.0442	**R^2**	0.2716
**Mean**	0.1621	**Adjusted R^2**	0.1969
**C.V. %**	27.24	**Predicted R^2**	0.0794
		**Adeq Precision**	5.9661

Final Equation in Terms of Actual Factors

1/Sqrt(Glu)	=
+0.201845	
-0.000676	H_2CO (37%)
+0.000571	CH_3OH
+5.01076E-06	H_2CO (37%) * CH_3OH
-5.80794E-06	CH_3OH^2

Table S9: ANOVA analysis of DoE settings. Significant model for asparagine (Asn).
Asn
Transform: Square Root
Constant: 0

Source	Sum of Squares	df	Mean Square	F-value	p-value	
**Model**	9.55	4	2.39	3.13	**0.0253**	significant
B- H_2CO (37%)	0.1416	1	0.1416	0.1854	0.6691	
C-CH_3OH	2.11	1	2.11	2.77	0.1041	
BC	3.01	1	3.01	3.94	0.0543	
C^2	4.41	1	4.41	5.78	**0.0211**	
**Residual**	29.79	39	0.7638			
Lack of Fit	6.54	10	0.6542	0.8162	0.6159	not significant
Pure Error	23.25	29	0.8016			
**Cor Total**	39.34	43				

Fit Statistics

**Std. Dev.**	0.8739	**R^2**	0.2429
**Mean**	1.68	**Adjusted R^2**	0.1652
**C.V. %**	51.92	**Predicted R^2**	0.0486
		**Adeq Precision**	5.7417

Final Equation in Terms of Actual Factors

Sqrt(Asn) =
+0.821300
+0.012061 H_2CO (37%)
-0.006891 CH_3OH
-0.000101 H_2CO (37%)* CH_3OH
+0.000099 CH_3OH^2

Table S10: ANOVA analysis of DoE settings. Significant model for serine (Ser).
Ser
Transform: Base 10 Log
Constant: 0

Source	Sum of Squares	df	Mean Square	F-value	p-value	
**Model**	1.18	2	0.5878	4.30	**0.0201**	significant
C-CH_3OH	0.2472	1	0.2472	1.81	0.1860	
C^2	0.9306	1	0.9306	6.81	**0.0126**	
**Residual**	5.60	41	0.1366			
Lack of Fit	1.80	12	0.1496	1.14	0.3685	not significant
Pure Error	3.81	29	0.1313			
**Cor Total**	6.78	43				

Fit Statistics

**Std. Dev.**	0.3696	**R^2**	0.1734
**Mean**	0.3264	**Adjusted R^2**	0.1331
**C.V. %**	113.24	**Predicted R^2**	0.0627
		**Adeq Precision**	6.1706

Final Equation in Terms of Actual Factors

$Log_{10}(Ser)$ =
+0.585712
-0.008698 CH_3OH
+0.000045 CH_3OH^2

Table S11: ANOVA analysis of DoE settings. Significant model for glycine (Gly).
Gly
Transform: Base 10 Log
Constant: 0

Source	Sum of Squares	df	Mean Square	F-value	p-value	
**Model**	1.24	4	0.3101	2.94	**0.0325**	significant
B- H_2CO (37%)2	0.0286	1	0.0286	0.2706	0.6059	
C-CH_3OH	0.2754	1	0.2754	2.61	0.1144	
BC	0.3612	1	0.3612	3.42	0.0720	
C^2	0.5880	1	0.5880	5.57	**0.0234**	
**Residual**	4.12	39	0.1056			
Lack of Fit	0.9094	10	0.0909	0.8219	0.6109	not significant
Pure Error	3.21	29	0.1106			
**Cor Total**	5.36	43				

Fit Statistics

**Std. Dev.**	0.3250	**R^2**	0.2315
**Mean**	1.56	**Adjusted R^2**	0.1527
**C.V. %**	20.78	**Predicted R^2**	0.0245
		**Adeq Precision**	5.5980

Final Equation in Terms of Actual Factors

$$
\begin{aligned}
\log_{10}(\text{Gly}) = \\
&+1.26420 \\
&+0.004282\ H_2CO\ (37\%) \\
&-0.002735\ CH_3OH \\
&-0.000035\ H_2CO\ (37\%)^*\ CH_3OH \\
&+0.000036\ CH_3OH^2
\end{aligned}
$$

Table S12: ANOVA analysis of DoE settings. Significant model for alanine (Ala).
Ala
Transform: Base 10 Log
Constant: 0

Source	Sum of Squares	df	Mean Square	F-value	p-value	
**Model**	0.8137	4	0.2034	2.77	**0.0408**	significant
B- H_2CO (37%)	0.0095	1	0.0095	0.1295	0.7209	
C-CH_3OH	0.1778	1	0.1778	2.42	0.1281	
BC	0.2269	1	0.2269	3.08	0.0869	
C^2	0.4099	1	0.4099	5.57	**0.0233**	
**Residual**	2.87	39	0.0736			
Lack of Fit	0.7646	10	0.0765	1.05	0.4266	not significant
Pure Error	2.10	29	0.0726			
**Cor Total**	3.68	43				

Fit Statistics

**Std. Dev.**	0.2712	**R^2**	0.2210
**Mean**	1.23	**Adjusted R^2**	0.1411
**C.V. %**	22.08	**Predicted R^2**	0.0077
		**Adeq Precision**	5.3901

Final Equation in Terms of Actual Factors

$Log_{10}(Ala)$ =
+1.01307
+0.003298 H_2CO (37%)
-0.002490 CH_3OH
-0.000028 H_2CO (37%)* CH_3OH
+0.000030 CH_3OH^2

Table S13: ANOVA analysis of DoE settings. Significant model for tyrosine (Tyr).
Tyr
Transform: Square Root
Constant: 0

Source	Sum of Squares	df	Mean Square	F-value	p-value	
**Model**	14.35	4	3.59	3.55	**0.0146**	significant
B- H_2CO (37%)	0.2950	1	0.2950	0.2917	0.5922	
C-CH_3OH	1.41	1	1.41	1.40	0.2444	
BC	3.77	1	3.77	3.73	0.0609	
C^2	8.96	1	8.96	8.86	**0.0050**	
**Residual**	39.44	39	1.01			
Lack of Fit	12.51	10	1.25	1.35	0.2534	not significant
Pure Error	26.94	29	0.9288			
**Cor Total**	53.80	43				

Fit Statistics

**Std. Dev.**	1.01	**R^2**	0.2668
**Mean**	1.68	**Adjusted R^2**	0.1916
**C.V. %**	59.76	**Predicted R^2**	0.0658
		**Adeq Precision**	5.6803

Final Equation in Terms of Actual Factors

$$\sqrt{Tyr} = +1.07569 + 0.013822\ H_2CO\ (37\%) - 0.015538\ CH_3OH - 0.000113\ H_2CO\ (37\%) * CH_3OH + 0.000141\ CH_3OH^2$$

Sqrt(Tyr) =
+1.07569
+0.013822 H_2CO (37%)
-0.015538 CH_3OH
-0.000113 H_2CO (37%)* CH_3OH
+0.000141 CH_3OH^2

Table S14: ANOVA analysis of DoE settings. Significant model for tryptophan (Trp).
Trp
Transform: Base 10 Log
Constant: 0

Source	Sum of Squares	df	Mean Square	F-value	p-value	
**Model**	1.40	4	0.3488	3.33	**0.0194**	significant
B- H_2CO (37%)	0.0360	1	0.0360	0.3430	0.5615	
C-CH_3OH	0.2690	1	0.2690	2.57	0.1172	
BC	0.4041	1	0.4041	3.86	0.0568	
C^2	0.6985	1	0.6985	6.66	**0.0137**	
**Residual**	4.09	39	0.1048			
Lack of Fit	0.8849	10	0.0885	0.8012	0.6287	not significant
Pure Error	3.20	29	0.1104			
**Cor Total**	5.48	43				

Fit Statistics

**Std. Dev.**	0.3237	**R^2**	0.2545
**Mean**	0.0349	**Adjusted R^2**	0.1780
**C.V. %**	927.81	**Predicted R^2**	0.0588
		**Adeq Precision**	5.8875

Final Equation in Terms of Actual Factors

$Log_{10}(Trp)$ =
-0.264752
+0.004557 H_2CO (37%)
-0.003246 CH_3OH
-0.000037 H_2CO (37%)* CH_3OH
+0.000039 CH_3OH^2

Table S15: ANOVA analysis of DoE settings. Significant model for phenylalanine (Phe).
Phe
Transform: Base 10 Log
Constant: 0

Source	Sum of Squares	df	Mean Square	F-value	p-value	
**Model**	0.7950	2	0.3975	3.72	**0.0327**	significant
C-CH_3OH	0.2231	1	0.2231	2.09	0.1560	
C^2	0.5737	1	0.5737	5.37	**0.0255**	
**Residual**	4.38	41	0.1068			
Lack of Fit	1.29	12	0.1078	1.01	0.4627	not significant
Pure Error	3.09	29	0.1064			
**Cor Total**	5.17	43				

Fit Statistics

**Std. Dev.**	0.3268	**R^2**	0.1536
**Mean**	0.8346	**Adjusted R^2**	0.1124
**C.V. %**	39.16	**Predicted R^2**	0.0463
		**Adeq Precision**	5.7348

Final Equation in Terms of Actual Factors

$$\log_{10}(\text{Phe}) = +1.01473 - 0.006613\ CH_3OH + 0.000036\ CH_3OH^2$$

Table S16: ANOVA analysis of DoE settings. Significant model for total amino acids.
Total Amino acids
Transform: Base 10 Log
Constant: 0

Source	Sum of Squares	df	Mean Square	F-value	p-value	
**Model**	1.01	4	0.2532	3.18	**0.0236**	significant
B- H_2CO (37%)	0.0344	1	0.0344	0.4325	0.5146	
C-CH_3OH	0.2325	1	0.2325	2.92	0.0954	
BC	0.2675	1	0.2675	3.36	0.0745	
C^2	0.4862	1	0.4862	6.11	**0.0180**	
**Residual**	3.11	39	0.0796			
Lack of Fit	0.8048	10	0.0805	1.01	0.4553	not significant
Pure Error	2.30	29	0.0793			
**Cor Total**	4.12	43				

Fit Statistics

**Std. Dev.**	0.2822	**R^2**	0.2459
**Mean**	2.24	**Adjusted R^2**	0.1686
**C.V. %**	12.61	**Predicted R^2**	0.0367
		**Adeq Precision**	5.8731

Final Equation in Terms of Actual Factors

$$
\begin{aligned}
\text{Log}_{10}(\text{Total Amino acids}) = \\
&+1.97501 \\
&+0.003791\ H_2CO\ (37\%) \\
&-0.002661\ CH_3OH \\
&-0.000030\ H_2CO\ (37\%)^*\ CH_3OH \\
&+0.000033\ CH_3OH^2
\end{aligned}
$$

Table S17: ANOVA analysis of DoE settings. Significant model for glutamine (Gln).

Gln
Transform: Square Root
Constant: 0

Source	Sum of Squares	df	Mean Square	F-value	p-value	
**Model**	4.36	2	2.18	4.57	**0.0162**	significant
A-NH_4Cl	2.85	1	2.85	5.96	**0.0190**	
C- CH_3OH	1.62	1	1.62	3.39	0.0727	
**Residual**	19.57	41	0.4772			
Lack of Fit	4.09	12	0.3411	0.6393	0.7916	not significant
Pure Error	15.47	29	0.5336			
**Cor Total**	23.92	43				

Fit Statistics

**Std. Dev.**	0.6908	**R^2**	0.1822
**Mean**	1.51	**Adjusted R^2**	0.1423
**C.V. %**	45.66	**Predicted R^2**	0.0593
		**Adeq Precision**	5.1932

Final Equation in Terms of Actual Factors

Sqrt(Gln) =
+2.42325
-0.071202 NH_4Cl
-0.003356 CH_3OH

Table S18: ANOVA analysis of DoE settings. Significant model for arginine (Arg).

Arg
Transform: Square Root
Constant: 0

Source	Sum of Squares	df	Mean Square	F-value	p-value	
**Model**	1.71	1	1.71	5.75	**0.0210**	significant
A-NH_4Cl	1.71	1	1.71	5.75	**0.0210**	
**Residual**	12.50	42	0.2976			
Lack of Fit	3.18	13	0.2450	0.7628	0.6900	not significant
Pure Error	9.31	29	0.3212			
**Cor Total**	14.21	43				

Fit Statistics

**Std. Dev.**	0.5455	**R^2**	0.1205
**Mean**	0.7682	**Adjusted R^2**	0.0995
**C.V. %**	71.01	**Predicted R^2**	0.0432
		**Adeq Precision**	5.9867

Final Equation in Terms of Actual Factors

Sqrt(Arg) =
+0.345122
+0.055201 NH_4Cl

Table S19: ANOVA analysis of DoE settings. Significant model for valine (Val).

Val
Transform: Inverse Sqrt
Constant: 0

Source	Sum of Squares	df	Mean Square	F-value	p-value	
**Model**	0.0563	1	0.0563	5.68	**0.0217**	significant
A-NH_4Cl	0.0563	1	0.0563	5.68	**0.0217**	
**Residual**	0.4159	42	0.0099			
Lack of Fit	0.1203	13	0.0093	0.9076	0.5561	not significant
Pure Error	0.2956	29	0.0102			
**Cor Total**	0.4722	43				

Fit Statistics

**Std. Dev.**	0.0995	**R^2**	0.1191
**Mean**	0.3033	**Adjusted R^2**	0.0982
**C.V. %**	32.81	**Predicted R^2**	0.0393
		**Adeq Precision**	5.9494

Final Equation in Terms of Actual Factors

1/Sqrt(Val) =
+0.380002
-0.010007 NH_4Cl

Table S20: ANOVA analysis of DoE settings. Significant model for isoleucine (Ile).

Ile
Transform: Base 10 Log
Constant: 0

Source	Sum of Squares	df	Mean Square	F-value	p-value	
**Model**	1.37	5	0.2741	3.13	**0.0183**	significant
A-NH_4Cl	0.4420	1	0.4420	5.05	**0.0305**	
B- H_2CO (37%)	0.0020	1	0.0020	0.0224	0.8819	
C- CH_3OH	0.2655	1	0.2655	3.04	0.0895	
BC	0.3051	1	0.3051	3.49	0.0695	
C^2	0.4161	1	0.4161	4.76	**0.0354**	
**Residual**	3.32	38	0.0875			
Lack of Fit	0.6451	9	0.0717	0.7760	0.6394	not significant
Pure Error	2.68	29	0.0924			
**Cor Total**	4.69	43				

Fit Statistics

**Std. Dev.**	0.2958	**R^2**	0.2919
**Mean**	1.08	**Adjusted R^2**	0.1988
**C.V. %**	27.38	**Predicted R^2**	0.0449
		**Adeq Precision**	5.9240

Final Equation in Terms of Actual Factors

$Log_{10}(Ile)$ =

+0.587403

+0.028086 NH_4Cl

+0.003644 H_2CO (37%)

-0.001805 CH_3OH

-0.000032 H_2CO (37%)* CH_3OH

+0.000030 CH_3OH^2

Table S21: ANOVA analysis of DoE settings. Significant model for leucine (Leu).

Leu
Transform: Base 10 Log
Constant: 0

Source	Sum of Squares	df	Mean Square	F-value	p-value	
**Model**	1.35	5	0.2700	3.04	**0.0210**	significant
A-NH4Cl	0.3884	1	0.3884	4.37	**0.0432**	
B- H_2CO (37%)	0.0024	1	0.0024	0.0273	0.8696	
C- CH_3OH	0.2784	1	0.2784	3.13	0.0847	
BC	0.3009	1	0.3009	3.39	0.0735	
C^2	0.4380	1	0.4380	4.93	**0.0324**	
**Residual**	3.37	38	0.0888			
Lack of Fit	0.6405	9	0.0712	0.7547	0.6573	not significant
Pure Error	2.73	29	0.0943			
**Cor Total**	4.73	43				

Fit Statistics

**Std. Dev.**	0.2980	**R^2**	0.2857
**Mean**	1.02	**Adjusted R^2**	0.1918
**C.V. %**	29.23	**Predicted R^2**	0.0391
		**Adeq Precision**	5.9139

Final Equation in Terms of Actual Factors

$Log_{10}(Leu)$ =

+0.544955

+0.026329 NH4Cl

+0.003633 H_2CO (37%)

-0.001971 CH_3OH

-0.000032 H_2CO (37%)* CH_3OH

+0.000031 CH_3OH^2

Table S22: ANOVA analysis of DoE settings. Significant model for threonine (Thr).

Thr
Transform: Square Root
Constant: 0

Source	Sum of Squares	df	Mean Square	F-value	p-value	
**Model**	6.73	4	1.68	3.21	**0.0227**	significant
B- H_2CO (37%)	0.7547	1	0.7547	1.44	0.2373	
C- CH_3OH	1.14	1	1.14	2.18	0.1478	
BC	2.25	1	2.25	4.29	**0.0450**	
C^2	2.57	1	2.57	4.90	**0.0328**	
**Residual**	20.44	39	0.5240			
Lack of Fit	5.39	10	0.5389	1.04	0.4376	not significant
Pure Error	15.05	29	0.5189			
**Cor Total**	27.17	43				

Fit Statistics

**Std. Dev.**	0.7239	**R^2**	0.2477
**Mean**	1.56	**Adjusted R^2**	0.1705
**C.V. %**	46.39	**Predicted R^2**	0.0220
		**Adeq Precision**	5.7448

Final Equation in Terms of Actual Factors

Sqrt(Thr) =
+0.620539
+0.011860 H_2CO (37%)
-0.004232 CH_3OH
-0.000087 H_2CO (37%)* CH_3OH
+0.000076 CH_3OH^2

Table S23: ANOVA analysis of DoE settings. Significant model for aspartic acid (Asp).

Asp
Transform: Inverse

Source	Sum of Squares	df	Mean Square	F-value	p-value	
**Model**	0.0117	2	0.0058	3.45	**0.0411**	significant
C- CH_3OH	0.0052	1	0.0052	3.06	0.0878	
C^2	0.0065	1	0.0065	3.86	0.0561	
**Residual**	0.0693	41	0.0017			
Lack of Fit	0.0200	12	0.0017	0.9778	0.4912	not significant
Pure Error	0.0493	29	0.0017			
**Cor Total**	0.0809	43				

Fit Statistics

**Std. Dev.**	0.0411	**R^2**	0.1441
**Mean**	0.1058	**Adjusted R^2**	0.1024
**C.V. %**	38.87	**Predicted R^2**	0.0280
		**Adeq Precision**	5.3969

Final Equation in Terms of Actual Factors

1/(Asp)	=
+0.092712	
+0.000649	CH_3OH
-3.81072E-06	CH_3OH^2

Table S24. Levene-tests (modified robust Brown-Forsythe Levene-type test based on the absolute deviations from the median) of lipid and amino acid production in the extreme values setting (non-significant result if p-value > 0.05, marked in bold). Abbreviations of amino acids: Asp = aspartic acid; Glu = glutamic acid; Asn = asparagine; Ser = serine; Gln = glutamine; His = histidine; Gly = glycine; Thr = threonine; Arg = arginine; Ala = alanine; Tyr = tyrosine; Val = valine; Met = methionine; Trp = tryptophan; Phe = phenylalanine; Ile = isoleucine; Leu = leucine.

**Lipids**		**Amino Acids**	
**ratio**	**p-value**	**species**	**p-value**
**Archaeol/macrocycle**	4.72e-07	**Asp**	**0.07071**
**Diethers/tetraethers**	**0.486**	**Glu**	0.001651
**GDGT/GMGT**	**0.2931**	**Asn**	2.315e-05
**GTGT/GDGT**	**0.1927**	**Ser**	0.0111
**GTGT/GMGT**	0.03613	**Gln**	0.04085
**GMGT-0/0′**	0.001651	**His**	n.a.
		**Gly**	0.007693
		**Thr**	0.01707
		**Arg**	0.0004998
		**Ala**	0.0007432
		**Tyr**	0.0118
		**Val**	0.005982
		**Met**	2.173e-05
		**Trp**	0.03481
		**Phe**	0.01328
		**Ile**	0.0002112
		**Leu**	0.002336
		**total**	0.008958

Table S25. ANOVA analysis of lipid and amino acid species that showed no significance at the performed Levene test (see Table S26, significant if Pr(>F) < 0.01). Asp = aspartic acid.

Analysis for Diethers/tetraethers

	Df	Sum Sq	Mean Sq	F value	Pr (>F)
**NH_4Cl**	1	0.112	0.112	0.0450	0.8354766
**H_2CO**	1	69.295	69.295	27.8926	**0.0001942**
**CH_3OH**	1	0.029	0.029	0.0119	0.9150552
**Residuals**	12	29.812	2.484		

Analysis for GDGT/GMGT

	Df	Sum Sq	Mean Sq	F value	Pr (>F)
**NH_4Cl**	1	0.4919	0.4919	4.1580	0.06409
**H_2CO**	1	7.7360	7.7360	65.3901	**3.368e-06**
**CH_3OH**	1	0.1326	0.1326	1.1209	0.31058
**Residuals**	12	1.4197	0.1183		

Analysis for GTGT-0/GDGT-0

	Df	Sum Sq	Mean Sq	F value	Pr (>F)
**NH_4Cl**	1	0.002138	0.002138	3.4467	0.08809
**H_2CO**	1	0.046884	0.046884	75.5676	**1.59e-06**
**CH_3OH**	1	0.000003	0.000003	0.0040	0.95034
**Residuals**	12	0.007445	0.000620		

Analysis for Asp

	Df	Sum Sq	Mean Sq	F value	Pr (>F)
**NH_4Cl**	1	197.030	197.030	11.0624	**0.006043**
**H_2CO**	1	5.556	5.556	0.3119	0.586766
**CH_3OH**	1	22.739	22.739	1.2767	0.280599
**Residuals**	12	213.730	17.811		

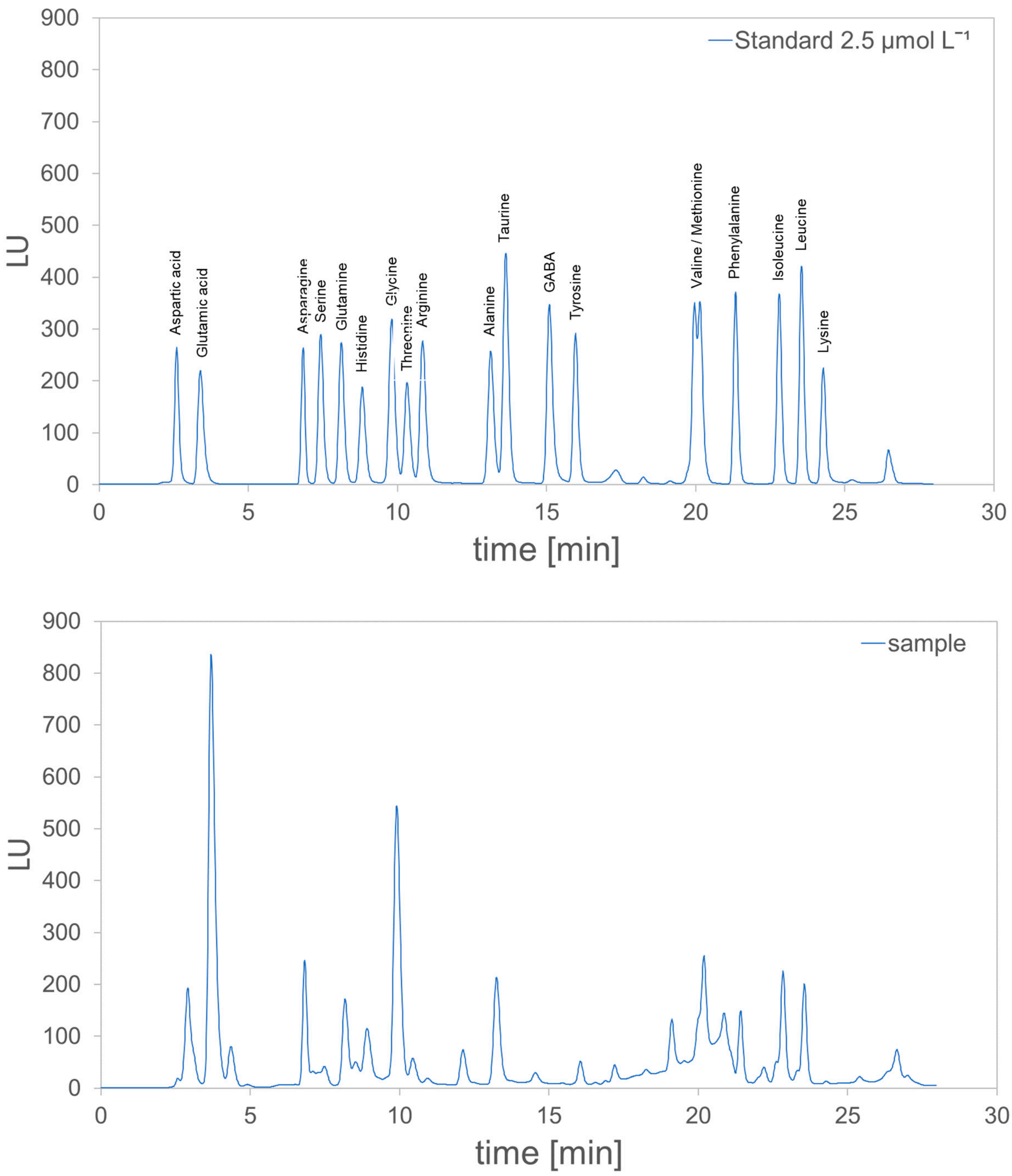

Figure S1. HPLC-Chromatograms of a 2.5 µmol L^{-1} standard (AAS18, Sigma Aldrich; the single amino acid tryptophane (Trp) is missing in that specific standard curve) in MQ-water compared with a sample (undiluted) analysed by the method published in Clifford *et al.*, 2017 [53]. Injection volume 500 µL, gain factor 10, method duration 28 min.

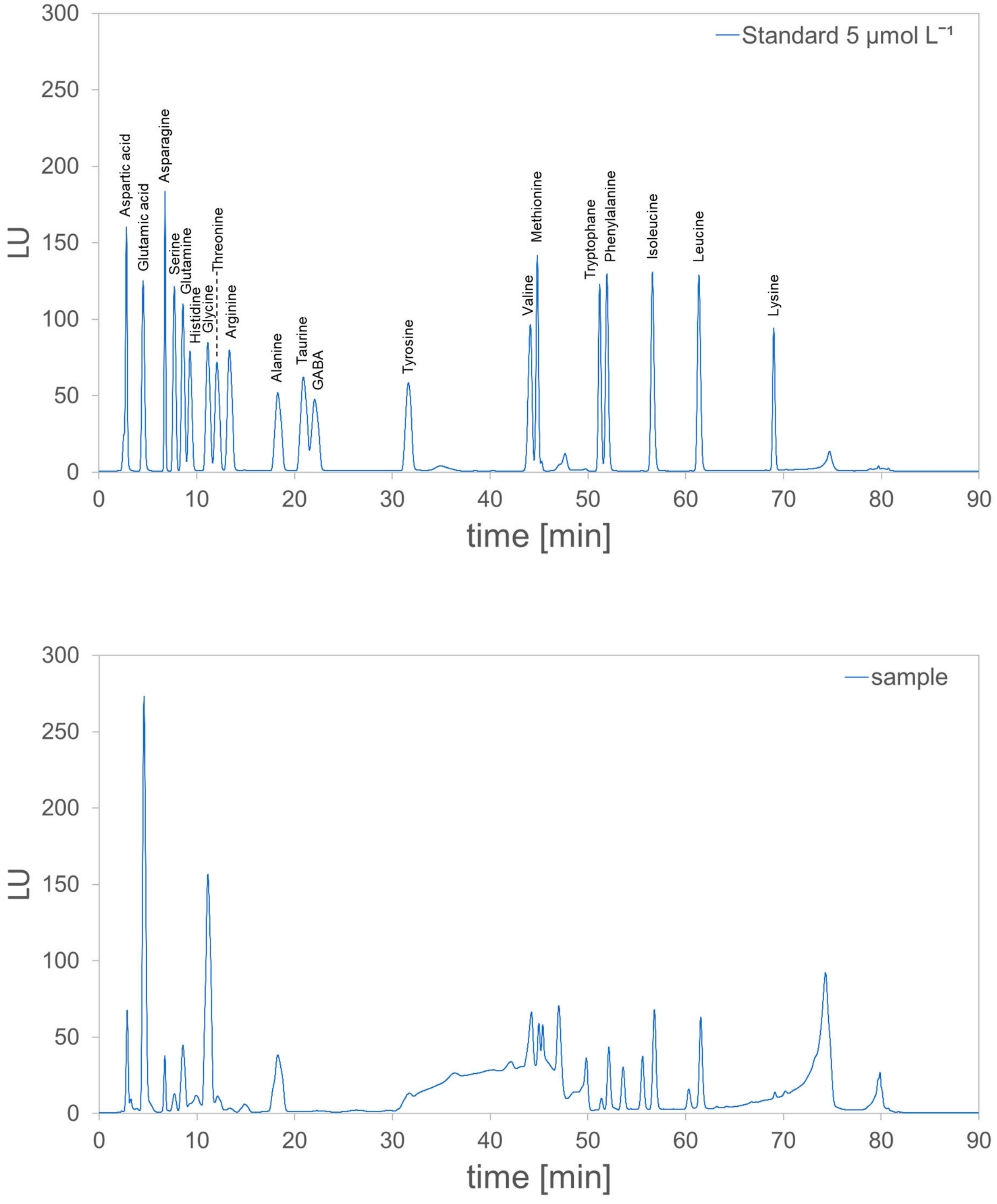

Figure S2. HPLC-Chromatograms of a 5 µmol L^{-1} standard (AAS18, Sigma Aldrich) in MQ-water compared with a sample (dilution 1:4, in MQ-water) analysed by the new method presented in this study. Injection volume 100 µL, gain factor 10, method duration 90 min.

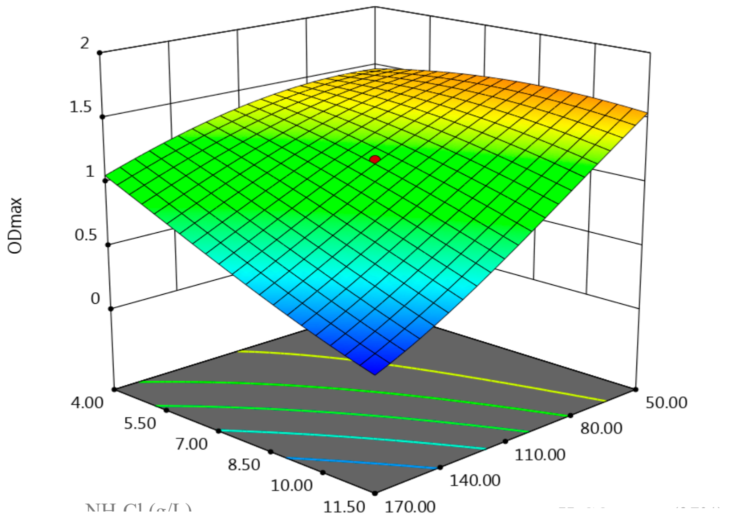

Figure S3. Results of the statistical analysis of the DoE. Significant dependences were calculated for OD_{max}.

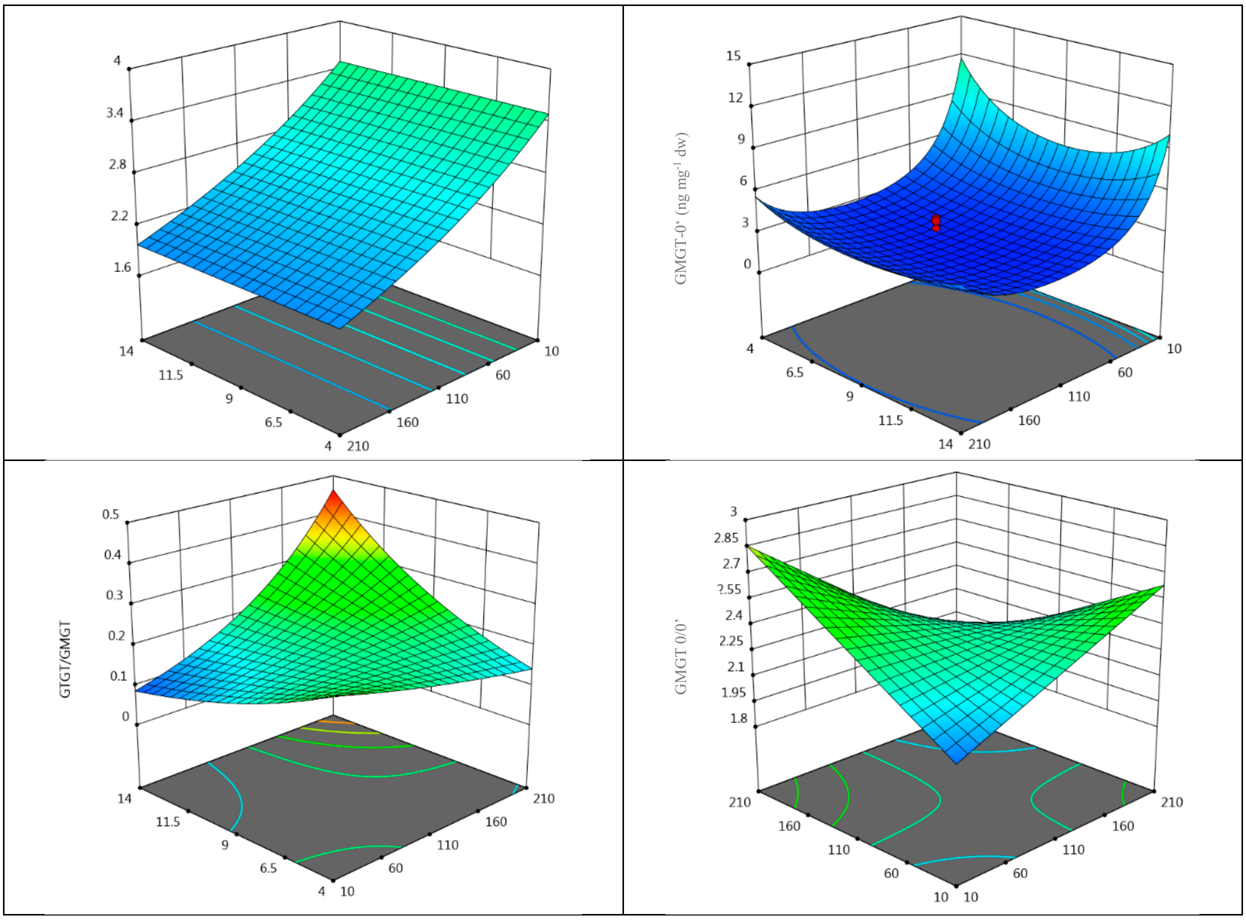

Figure S4. Results of the statistical analysis of the DoE. Significant dependences were calculated for GTGT-0, GMGT-0′, GTGT/GMGT (including both GMGT isomers), and GMGT-0/0′.

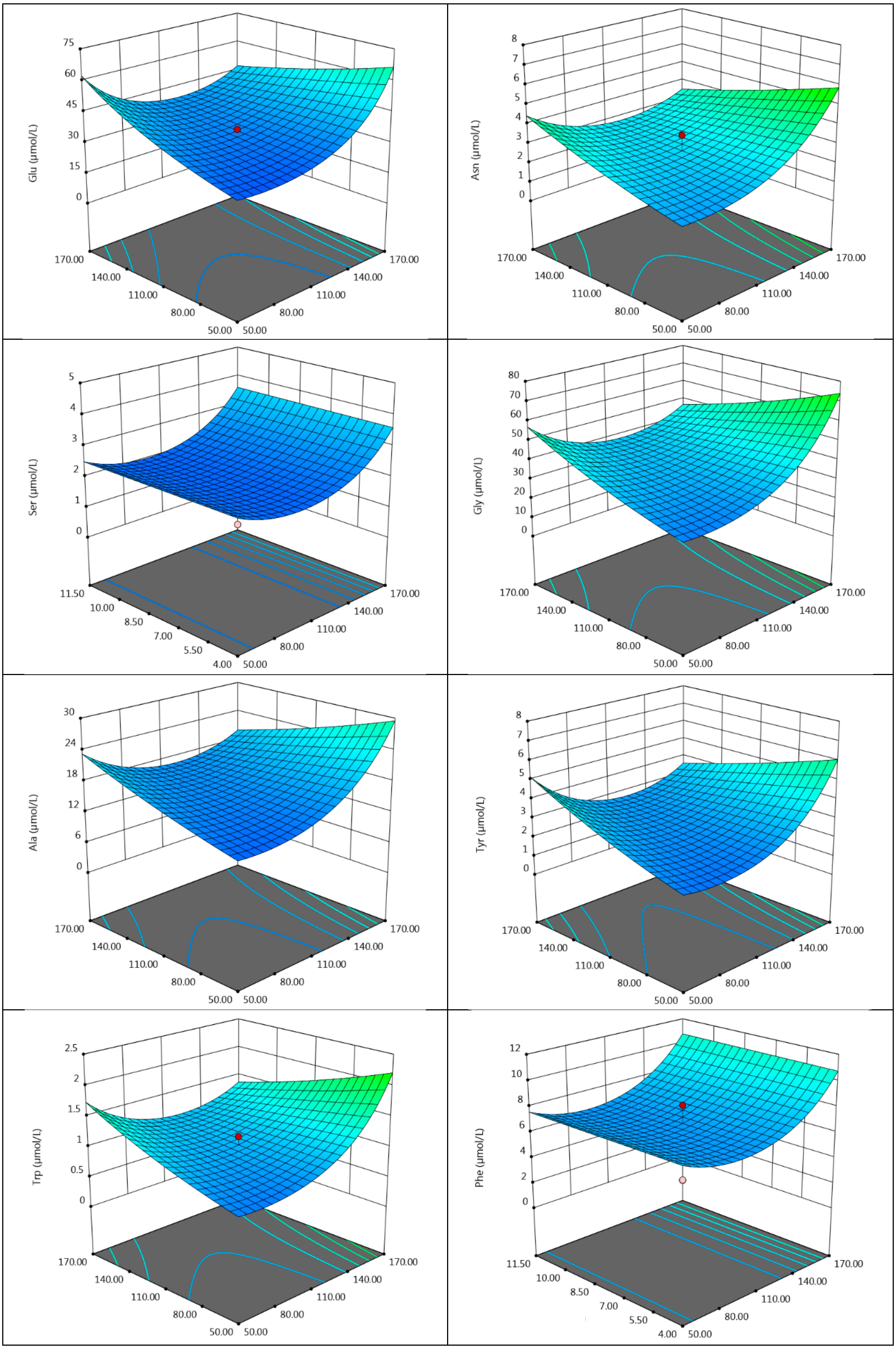
Glu (μmol/L)
75
60
45
30
15
0
170.00
140.00
110.00
80.00
50.00
50.00
80.00
110.00
140.00
170.00
Asn (μmol/L)
8
7
6
5
4
3
2
1
0
170.00
140.00
110.00
80.00
50.00
50.00
80.00
110.00
140.00
170.00
Ser (μmol/L)
5
4
3
2
1
0
11.50
10.00
8.50
7.00
5.50
4.00
50.00
80.00
110.00
140.00
170.00
Gly (μmol/L)
80
70
60
50
40
30
20
10
0
170.00
140.00
110.00
80.00
50.00
50.00
80.00
110.00
140.00
170.00
Ala (μmol/L)
30
24
18
12
6
0
170.00
140.00
110.00
80.00
50.00
50.00
80.00
110.00
140.00
170.00
Tyr (μmol/L)
8
7
6
5
4
3
2
1
0
170.00
140.00
110.00
80.00
50.00
50.00
80.00
110.00
140.00
170.00
Trp (μmol/L)
2.5
2
1.5
1
0.5
0
170.00
140.00
110.00
80.00
50.00
50.00
80.00
110.00
140.00
170.00
Phe (μmol/L)
12
10
8
6
4
2
0
11.50
10.00
8.50
7.00
5.50
4.00
50.00
80.00
110.00
140.00
170.00

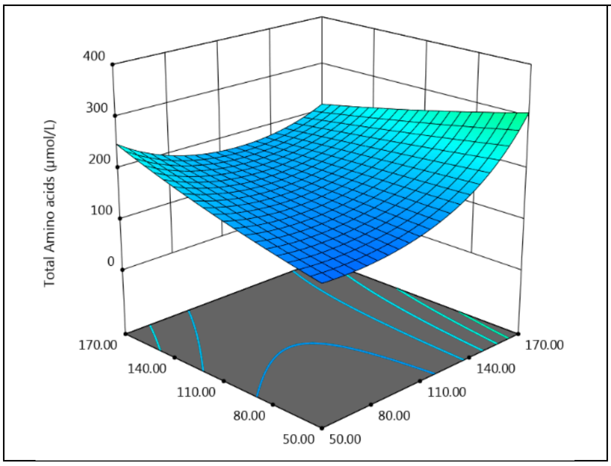

Figure S5. Glu (glutamic acid), Asn (asparagine), Ser (serine), Gly (glycine), Ala (alanine), Tyr (tyrosine), Trp (tryptophan), Phe (phenylalanine), and total amino acids show a dependence on CH_3OH squared.

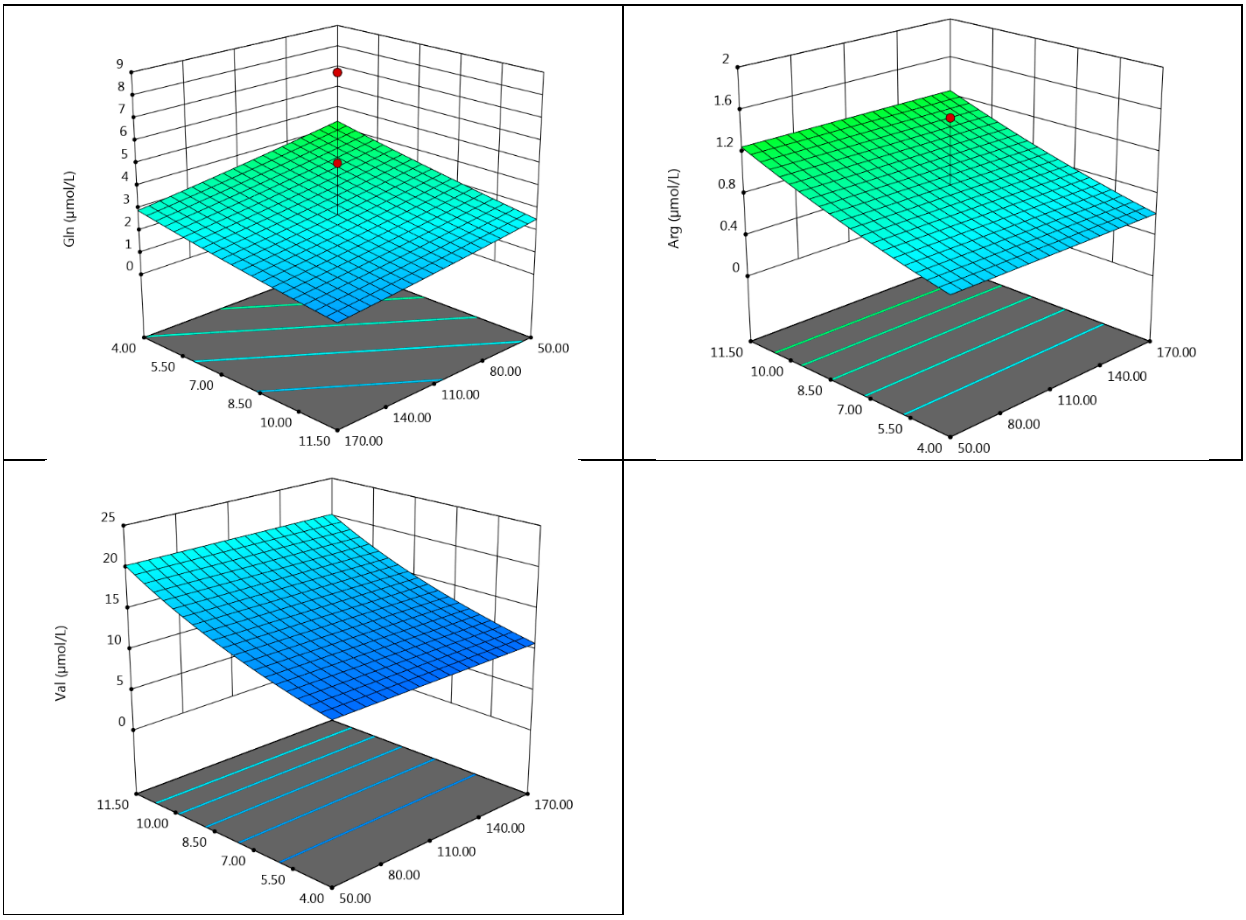

Figure S6. Gln (glutamine), Arg (arginine), and Val (valine) are influenced by the presence of NH_4Cl.

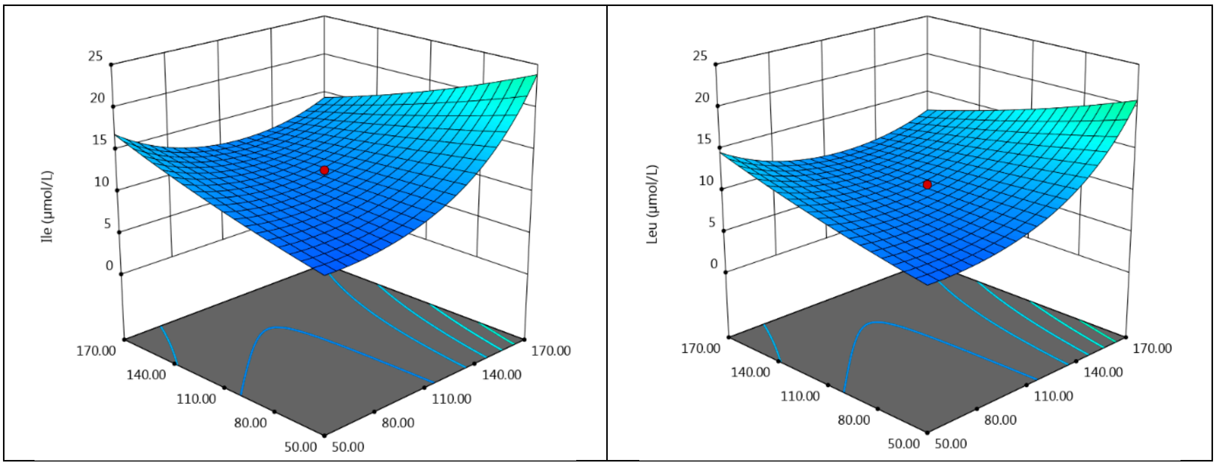

Figure S7. Ile (isoleucine) and Leu (leucine) are characterized by the influence of NH_4Cl and CH_3OH squared.

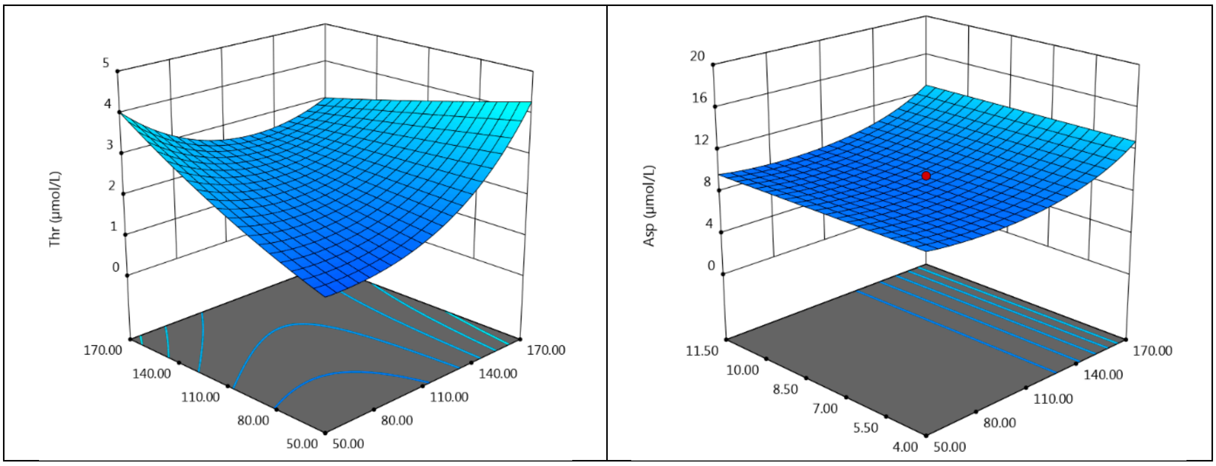

Figure S8. Thr (threonine) is influenced by the interaction of CH_3OH and H_2CO and CH_3OH squared, and Asp (aspartic acid) shows no specific dependency at all.

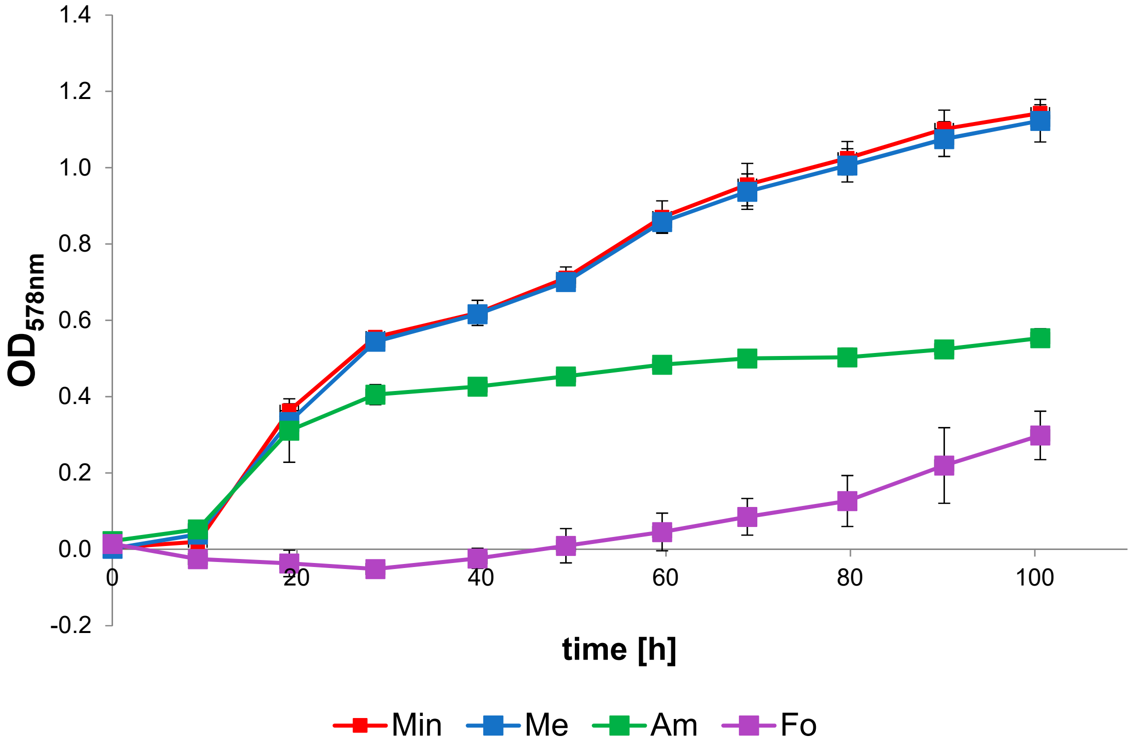

Figure S9. Average growth curves of the four different experiments (n=4, the OD of the respective zero control was subtracted, error bars present respective standard deviations).

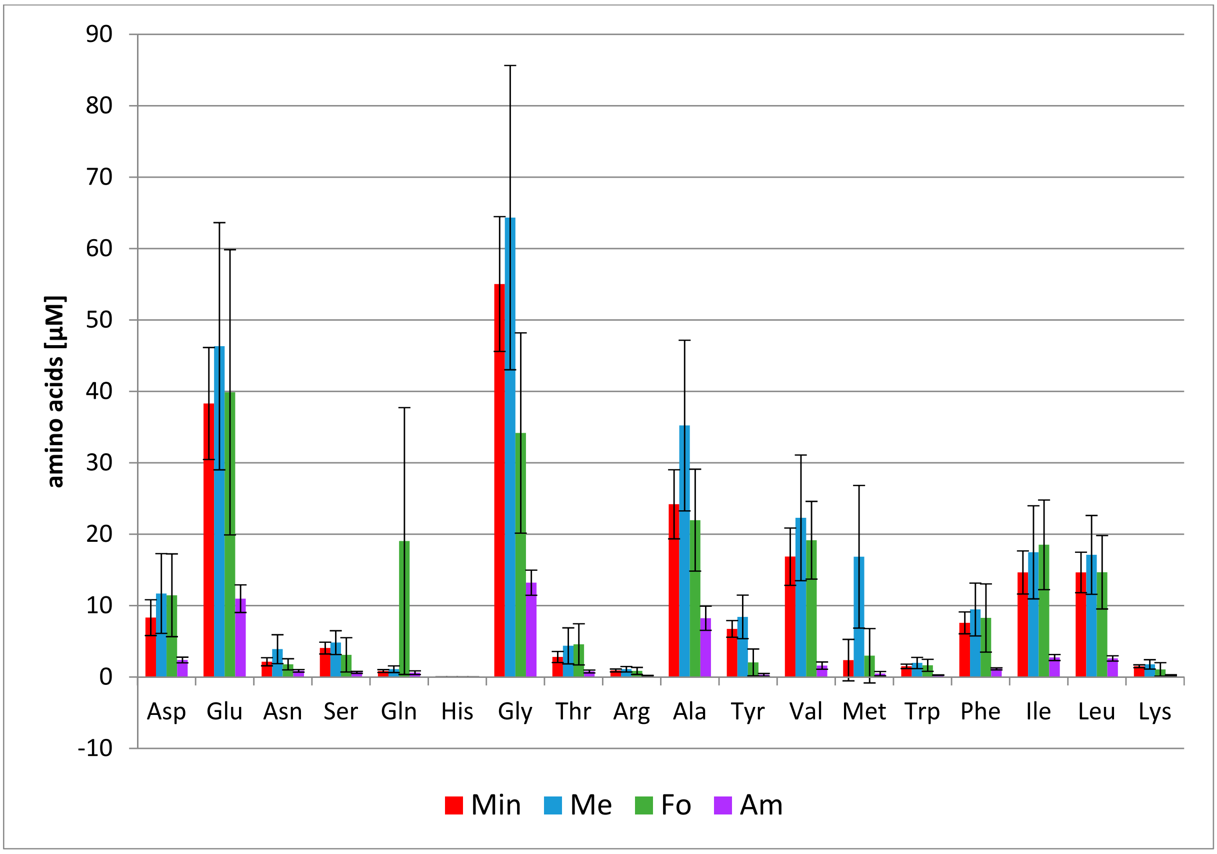

Figure S10. Concentrations of the different amino acids for the four different experimental settings (n=4, error bars present respective standard deviations). The high error of Gln for the "Fo"-setting is caused by a wide spreading of the data points between 4 and 46 µM. However, even the error is high, all single samples of the "Fo"-set showed a value far higher in Gln than in any other sample. Abbreviations of amino acids: Asp = aspartic acid; Glu = glutamic acid; Asn = asparagine; Ser = serine; Gln = glutamine; His = histidine; Gly = glycine; Thr = threonine; Arg = arginine; Ala = alanine; Tyr = tyrosine; Val = valine; Met = methionine; Trp = tryptophan; Phe = phenylalanine; Ile = isoleucine; Leu = leucine; Lys = lysine.
